# Recurrent chromosomal gains and heterogeneous driver mutations characterise papillary renal cancer evolution

**DOI:** 10.1038/ncomms7336

**Published:** 2015-03-19

**Authors:** Michal Kovac, Carolina Navas, Stuart Horswell, Max Salm, Chiara Bardella, Andrew Rowan, Mark Stares, Francesc Castro-Giner, Rosalie Fisher, Elza C. de Bruin, Monika Kovacova, Maggie Gorman, Seiko Makino, Jennet Williams, Emma Jaeger, Angela Jones, Kimberley Howarth, James Larkin, Lisa Pickering, Martin Gore, David L. Nicol, Steven Hazell, Gordon Stamp, Tim O’Brien, Ben Challacombe, Nik Matthews, Benjamin Phillimore, Sharmin Begum, Adam Rabinowitz, Ignacio Varela, Ashish Chandra, Catherine Horsfield, Alexander Polson, Maxine Tran, Rupesh Bhatt, Luigi Terracciano, Serenella Eppenberger-Castori, Andrew Protheroe, Eamonn Maher, Mona El Bahrawy, Stewart Fleming, Peter Ratcliffe, Karl Heinimann, Charles Swanton, Ian Tomlinson

**Affiliations:** 1 Molecular and Population Genetics Laboratory, Wellcome Trust Centre for Human Genetics, Nuffield Department of Clinical Medicine, University of Oxford, Roosevelt Drive, Oxford OX3 7BN, UK; 2 Department of Biomedicine, Research Group Human Genomics, University of Basel, Mattenstrasse 28, 4058 Basel, Switzerland; 3 Translational Cancer Therapeutics Laboratory, London Research Institute, Cancer Research UK, 44, Lincoln’s Inn Fields, London WC2A 3LY, UK; 4 Bioinformatics and Biostatistics, London Research Institute, Cancer Research UK, 44, Lincoln’s Inn Fields, London WC2A 3LY, UK; 5 University College London Cancer Institute and Hospitals, Huntley Street, London WC1E 6DD, UK; 6 Faculty of Mechanical Engineering, Institute of Mathematics and Physics, Slovak University of Technology, Namestie slobody 17, 812 31 Bratislava, Slovakia; 7 Department of Medicine, The Royal Marsden NHS Foundation Trust, 203 Fulham Road, London SW3 6JJ, UK; 8 Department of Urology, The Royal Marsden NHS Foundation Trust, 203 Fulham Road, London SW3 6JJ, UK; 9 School of Medicine, University of Queensland, Brisbane, Australia; 10 Department of Histopathology, The Royal Marsden NHS Foundation Trust, 203 Fulham Road, London SW3 6JJ, UK; 11 Experimental Histopathology, London Research Institute, Cancer Research UK, 44, Lincoln’s Inn Fields, London WC2A 3LY, UK; 12 Urology Centre, Guy’s and St Thomas’s Hospital NHS Foundation Trust, Great Maze Pond, London SE1 9RT, UK; 13 Advanced Sequencing Laboratory, London Research Institute, Cancer Research UK, 44, Lincoln’s Inn Fields, London WC2A 3LY, UK; 14 Genomic analysis of tumour development, Instituto de Biomedicina y Biotecnología de Cantabria (CSIC-UC-Sodercan), Departamento de Biología Molecular, Universidad de Cantabria, 39011 Santander, Spain; 15 Department of Histopathology, Guy’s and St Thomas’s Hospital NHS Foundation Trust, Great Maze Pond, London SE1 9RT, UK; 16 Department of Oncology, Uro-Oncology Research Group, University of Cambridge, Cambridge CB2 0RE, UK; 17 Department of Urology, University Hospitals, Birmingham B15 2TH, UK; 18 Institute for Pathology, University Hospital Basel, Schönbeinstrasse 40, 4003 Basel, Switzerland; 19 Department of Oncology, Cancer and Haematology Centre, Churchill Hospital, Oxford University Hospitals, Oxford OX3 7LJ, UK; 20 Department of Medical Genetics, University of Cambridge, Cambridge CB2 0QQ, UK; 21 Department of Histopathology, Imperial College London, Hammersmith Hospital, London W12 0HS, UK; 22 Department of Histopathology, Medical Research Institute, University of Dundee Medical School, Ninewells Hospital, Dundee DD1 9SY, UK; 23 Hypoxia Biology Laboratory, Henry Wellcome Building for Molecular Physiology, Nuffield Department of Clinical Medicine, University of Oxford, Roosevelt Drive, Oxford OX3 7BN, UK; 24 NIHR Comprehensive Biomedical Research Centre, University of Oxford, Roosevelt Drive, Oxford OX3 7BN, UK

## Abstract

Papillary renal cell carcinoma (pRCC) is an important subtype of kidney cancer with a problematic pathological classification and highly variable clinical behaviour. Here we sequence the genomes or exomes of 31 pRCCs, and in four tumours, multi-region sequencing is undertaken. We identify *BAP1*, *SETD2*, *ARID2* and Nrf2 pathway genes (*KEAP1*, *NHE2L2* and *CUL3*) as probable drivers, together with at least eight other possible drivers. However, only ~10% of tumours harbour detectable pathogenic changes in any one driver gene, and where present, the mutations are often predicted to be present within cancer sub-clones. We specifically detect parallel evolution of multiple *SETD2* mutations within different sub-regions of the same tumour. By contrast, large copy number gains of chromosomes 7, 12, 16 and 17 are usually early, monoclonal changes in pRCC evolution. The predominance of large copy number variants as the major drivers for pRCC highlights an unusual mode of tumorigenesis that may challenge precision medicine approaches.

## Abstract (web summary)

Papillary renal cell carcinoma (pRCC) is a subtype of kidney cancer characterized by highly variable clinical behaviour. Here the authors sequence either the genomes or exomes of 31 pRCCs and identify several genes in sub-clones and large copy number variants in major clones that may be important drivers of pRCC.

## Graphical abstract

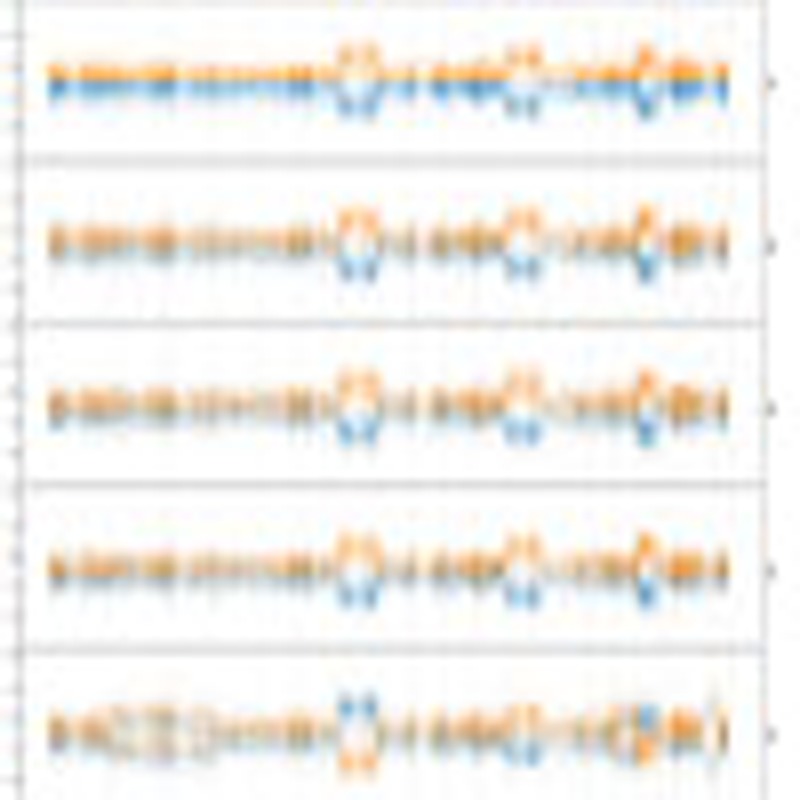

Primary renal cell cancer (RCC) constitutes about 2% of the cancers in western populations and tumours show a wide range of clinical behaviours. The most common type of RCC has a clear cell morphology (ccRCC) and usually arises owing to mutations in the *VHL* tumour suppressor gene^1^. Several other ccRCC driver mutations have been identified^2^. Papillary RCC (pRCC) is the second most common morphological type and germline *MET*, *FH* and, occasionally, *FLCN* mutations predispose to pRCCs. These mutations respectively cause the Mendelian conditions of hereditary pRCC^3^, hereditary leiomyomatosis and RCC^4^, and Birt-Hogg-Dubé syndrome^5^. Somatic *MET* mutations occur in a small proportion of sporadic pRCCs and a greater number show somatic copy number gains involving the *MET* locus on chromosome 7q^3^. Rarer RCC subtypes include collecting duct carcinoma, oncocytoma and chromophobe carcinoma. The genetic basis of these less common lesions is complex, and includes germline *FLCN* mutations in some cases.

RCCs resulting from high-penetrance germline mutations are frequently multi-focal, but up to 20% of apparently sporadic RCCs also have several discrete nodules in one or both kidneys. Clonality analysis of multiple nodules from sporadic ccRCCs has historically used microsatellite-based loss of heterozygosity (LOH), and these analyses have mostly concluded that ccRCCs are monoclonal. Other analyses of multi-focal, sporadic pRCCs have, by contrast, shown polyclonal origins of each nodule^6^,^7^,^8^. Recent studies based on next-generation sequencing have largely resolved the issue of ccRCC clonality, showing a common initiating *VHL* mutation, followed by divergence as different subsequent driver mutations are acquired and selected^9^. However, no comparable analysis of multi-focal pRCCs has previously been performed, and it remains possible that the nodules of these pRCCs have truly independent (epi)genetic origins.

pRCCs comprise about 10–15% or all RCCs and are thought to develop from the proximal or distal convoluted tubule^10^. They are sub-divided into two histological types. Type 1 lesions typically contain small cuboidal cells and thin papillae, with small, uniform nuclei and basophilic cytoplasm. Type 2 tumours have larger cells with eosinophilic cytoplasm and pseudostratification. However, mixed tumours are relatively common, and the classification is challenging and hence not universally adopted. Several studies have identified recurrent chromosomal copy number changes in pRCCs, the most consistent being gain of chromosome 7, 12, 16, 17 and 20, and loss of 6q and X^11^,^12^,^13^,^14^,^15^,^16^,^17^. Type 1 and 2 cancers have been reported as having different copy number profiles, but the data vary considerably among studies, perhaps as a result of inconsistencies in morphological assignment. In contrast to ccRCC^18^, relatively little is known about the mutations that drive pRCC growth and the clonality of copy number events and single-nucleotide variants (SNVs), apart from the small minority of cancers with changes in *MET* and *FH.*

In this study, we aim to search for driver mutations and recurrent copy number events and decipher the evolutionary landscape of pRCC, by inferring clonality from both single- and multi-region sampling strategies. We find driver mutations in *BAP1*, *SETD2*, *ARID2* and Nrf2 pathway genes that frequently occur within sub-clones, and recurrent, large-scale copy number changes that are usually present in major clones.

## Results

### Overview of the pRCC exome

Our discovery set comprised 31 pRCCs and paired normal tissue or peripheral blood (Table 1). Three tumours were separate foci from case GK116; these appeared to be grossly distinct lesions and were regarded as such for sequence analysis. All tumours were therapy naive, with the exception of patient RK36 who had been treated with sunitinib before cancer resection. Twenty of our pRCCs had type 2 morphology, the remaining 11 having type 1 morphology or mixed features.

Nineteen tumours (P01-07, P16-23, GK116_2, GK116_3, GK102 and RK133) underwent Agilent SureSelect or Illumina TruSeq exome capture, followed by sequencing on the Illumina HiSeq platform at median 110 × depth (cross-sample range 27–372 × ). A further four cancers (GK101, GK116_1, RK30 and RK36) underwent multi-region sampling, SureSelect exome capture and Illumina HiSeq sequencing, but the sequence data from the multiple regions of these tumours were combined for our initial analyses, resulting in median 173 × read depth (cross-sample range 131–240 × ). The remaining eight pRCCs (P08-15) underwent whole-genome sequencing using the Complete Genomics platform^19^. Further details of basic sequencing performance parameters and technical validation are given in the Supplementary Methods.

**Figure 1:**
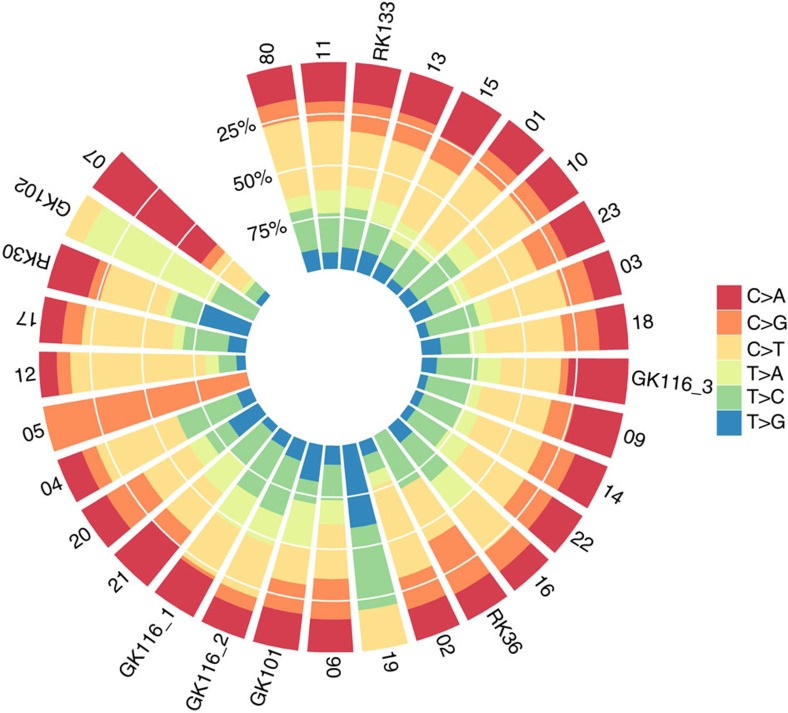
Somatic SNV spectra. Data are derived from the exome of each cancer. For the multi-region cancers, only the region with the highest mutation burden is displayed to provide a comparison with the other tumours. Note that cancers with very few somatic SNVs are shown for the sake of completeness.

The exonic SNV spectrum for each cancer is shown in Fig. 1 and Supplementary Table 1. C:G>T:A changes were the most common, followed by T:A>C:G, C:G>A:T and C:G>G:C changes. Seven cancers showed significant deviation (*q*_weighted_<0.05) from the global mutation spectrum (Supplementary Table 1). The outlying cancers tended to have higher mutation burdens, suggesting a specific genomic instability or mutagen exposure. Of the four most extreme cancers, two (GK102, P17) had a large proportion of C:G>T:A changes, one (P07) tended to acquire C:G>A:T changes and another (RK30) had mostly T:A>G:C changes. In no case did we identify a specific cause of genome instability, such as a mutant DNA repair gene, and no examples of kataegis were found. The genome-wide SNV mutation spectra for the Complete Genomics samples closely resembled those of the samples with exome sequence data.

Mutation signatures based on 95 otherwise unpublished TCGA pRCC exomes have been reported by Alexandrov *et al*^20^ who identified signature 5, and to a lesser extent 2 (‘APOBEC’), as prevalent. Signature 5 is characterized by a small excess of C>T and T>C changes of uncertain aetiology with no clear trinucleotide context bias. With the exception of the relatively hypermutant cancers highlighted above, most of our pRCCs’ mutation spectra were consistent with signature 5 (Supplementary Data 1). Signature 2 is characterized by C>T and C>G changes, especially when the preceding base is T, but none of our cancers clearly had this signature. Cancer P12 was notable, in that it did not have a high mutation burden, but had a broad excess of C>T changes, especially when the following base was A or G.

Twenty-three cancers were analysed for copy number changes and LOH using Affymetrix single-nucleotide polymorphism (SNP) arrays. Large somatic copy number changes (>30% chromosome arm) were present in 18/23 genomes (Table 1), with a total of 105 changes (median=5 and range 0–12). There was evidence of bimodality in the number of large somatic copy number alterations (SCNAs) (Supplementary Fig. 2). SCNA burden did not correlate with any of the global SNV metrics (details not shown). Gain of copy number was about twice as frequent as deletions and copy-neutral LOH was uncommon. We examined chromosomes for profound, complex rearrangements suggestive of chromothripsis^21^ or chromoplexy^22^. Two of the 23 cancers (P09 and P23) showed such rearrangement (Supplementary Fig. 3) on three and one chromosomes, respectively. Notably, in both cases, chromosome 2 was involved and the patterns of rearrangements on this chromosome were remarkably similar between the two cancers.

### Identification of pRCC driver genes

We initially investigated all 31 cancers and paired constitutional DNA for mutations in the Mendelian pRCC genes *MET*, *FH* and *FLCN* (Supplementary Table 2). Patient P11 carried the germline *FH* variant c.1189G>A (p.Gly397Arg) and the tumour showed copy-neutral LOH around *FH*. Functional prediction programmes and the *FH* mutation database reported this variant as likely to be pathogenic ( http://chromium.liacs.nl/lovd_sdh/variants.php?action=search_unique&select_db=FH). No somatic *FH* mutations were found. Three germline *MET* variants of uncertain significance were found (Supplementary Table 2). Tumour RK36 had acquired a somatic *MET* mutation, c.G799A (p.Glu267Lys; Table 2) that has strong predicted functional effects and lies next to a residue recurrently mutated in human cancer, but has not itself been reported as mutated (http://cancer.sanger.ac.uk/cosmic/gene/overview?ln=MET). No germline *FLCN* mutations were present, although the cancer of patient P15 carried a somatic, protein-truncating mutation (c.1568_1569insG, p.Lys523fs) in the last exon of *FLCN* (Table 2). This mutation was not accompanied by LOH.

**Figure 2:**
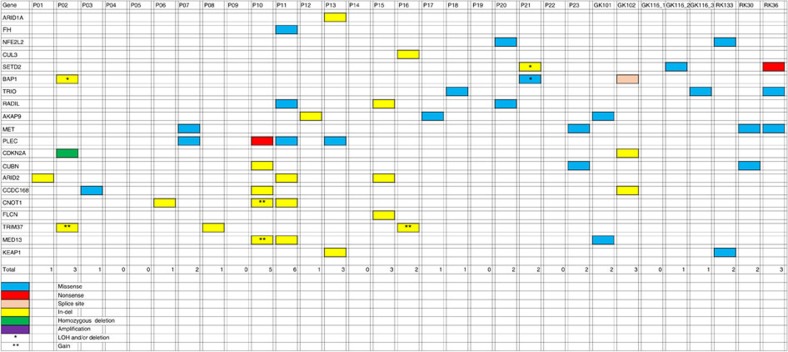
Distribution of selected somatic SNVs with predicted pathogenic effects and indels across cancers. The germline *FH* mutation, the somatic *CDKN2A* deletion and the large deletion with break point within *ARID1A* are also shown for completeness. Note that copy number and LOH data are not shown for cancers GK101, GK102, GK116_1, GK116_2, GK116_3, RK133, RK30 and RK36 since these lack SNP array data.

For discovery of somatic pRCC driver mutations, we examined somatic mutation calls from the 31 cancers with exome- and genome-sequence data, and restricted our analysis to protein-coding regions. A minimum variant allele frequency of 0.05 was set. We then prioritized genes for further investigation by filtering out somatic mutations to exclude all SNVs with benign predicted functional effects (SIFT score>0.3 and Polyphen2 score<0.7). All protein-truncating and splice-site mutations were retained. We identified genes that were somatically mutated in three or more cancers and inspected all mutant sequencing reads in the Integrated Genome Viewer to exclude any mutations at sites of evidently poor sequencing quality. Twelve genes (*SETD2*, *BAP1*, *TRIO*, *RADIL*, *AKAP9*, *PLEC*, *CUBN*, *ARID2*, *CCDC168*, *CNOT1*, *TRIM37* and *MED13*) remained after this filtering process (Table 2; Fig. 2).

We chose three genes—*SETD2*, *BAP1* and *ARID2*—for replication testing on the basis of their known or potential functional roles and the presence of at least one protein-truncating mutation. *SETD2* and *BAP1* both lie on the short arm of chromosome 3, undergo copy-neutral LOH or deletion (Fig. 2) and are driver genes for ccRCC and other cancer types. SETD2 is a histone methyltransferase and BAP1 is a deubiquitinase involved in the control of polycomb repressors^18^,^23^. ARID2 is a subunit of the PBAF chromatin-remodelling complex, has been reported as a potential driver gene in hepatocellular carcinoma^24^ and is functionally related to other cancer genes including *ARID1A*. Following independent confirmation of the *SETD2*, *BAP1* and *ARID2* mutations in our discovery phase samples using Sanger or Ion Torrent sequencing, we screened a replication set of 60 archival pRCCs for mutations in these genes (Supplementary Table 3), resulting in an additional five *BAP1*, three *SETD2*, and three *ARID2* mutations. Most of these mutations had good evidence of being pathogenic, based on prediction programs, conservation, presence of deletion/LOH, mutation reports in other cancer types and previous functional studies. Further details of the functional annotation of the *BAP1*, *SETD2* and *ARID2* mutations, together with additional information on our search for new driver mutations, are given in Supplementary Table 4.

Since there was pre-existing functional evidence for the importance of the stress response mediator Nrf2 in the pathogenesis of pRCCs^25^,^26^ and a small independent study (five pRCC exomes) had provided limited support for this notion^27^, we performed a focussed examination of three members of the Nrf2 pathway for somatic mutations: *NFE2L2*, which encodes Nrf2; *CUL3*, an Nrf2 ubiquitin ligase; and *KEAP1*, the Nrf2-specific ubiquitin ligase adaptor. We found *KEAP1* mutations in two cancers (Table 2). One tumour (P13) had acquired a frameshift change and another (RK133) carried a somatic SNV, p.Arg320Gly, of probable pathogenicity (SIFT=0.03 and Polyphen2=0.99) at an evolutionarily residue that is recurrently mutated in lung cancers (http://cancer.sanger.ac.uk/cosmic/gene/analysis?ln=KEAP1; http://www.cbioportal.org/public-portal/cross_cancer.do?cancer_study_id=all&data_priority=1&case_ids=&gene_set_choice=user-defined-list&gene_list=NFE2L2%0D%0AKEAP1&clinical_param_selection=null&tab_index=tab_visualize&Action=Submit#crosscancer/overview/1/NFE2L2%20KEAP1). A protein-truncating *CUL3* mutation was found in one cancer (P16). Missense *NFE2L2* mutations of predicted functionality p.Asp77Ala (SIFT=0.15 and Polyphen2=1.00) and p.Leu30Phe (SIFT=0.00 and Polyphen2=1.00) were present in tumours P20 and RK133, respectively (Table 2); both these specific changes were at evolutionarily conserved sites and had been reported previously as very probably pathogenic in lung carcinomas (http://cancer.sanger.ac.uk/cosmic/gene/analysis?ln=NFE2L2; http://www.cbioportal.org/public-portal/cross_cancer.do?cancer_study_id=all&data_priority=1&case_ids=&gene_set_choice=user-defined-list&gene_list=KEAP1%0D%0ANFE2L2&clinical_param_selection=null&tab_index=tab_visualize&Action=Submit#crosscancer/overview/1/KEAP1%20NFE2L2).

We then examined publicly available TCGA pRCC data (https://tcga-data.nci.nih.gov/tcga/tcgaCancerDetails.jsp?diseaseType=KIRP&diseaseName=Kidney%20renal%20papillary%20cell%20carcinoma) for somatic SNVs in our 20 candidate pRCC drivers (Fig. 2), using the Intogen^28^ and MutSigCV^29^ programs to identify significantly mutated genes (Supplementary Table 5). This analysis confirmed *SETD2*, *BAP1*, *NFE2L2* and *CUL3* as drivers, with a more modest degree of support for some other genes, including *MET*, *ARID1A*, *ARID2* and, interestingly, *TRIO.* Of note, no gene predicted by Intogen to be a driver at q=0.05 was mutated in >10% of all TCGA samples, confirming our finding that high-frequency driver SNVs are not present in pRCC.

### Structural and copy number mutation analysis

Global SCNA data are summarized in Supplementary Fig. 4. The most common changes were gains involving chromosomes 17q (*N*=14), 7 (*N*=11), 16 (*N*=10) and 12 (*N*=7). These changes tended to co-occur (*P*=0.026, exact binomial test). It has been plausibly suggested that gain of chr7 in pRCC targets *MET*^30^,^31^, and in the 23 pRCCs with SNP array data, we examined SCNAs for evidence that specific genes were targeted. However, in our cancers, chr7 gain always involved the whole chromosome. Similarly, all the gains of chromosomes 12, 16 and 17 involved large regions (minimal regions shown in Supplementary Table 6).

Large deletions were about half as common as gains, the most frequent involving chromosomes 3p (*N*=5), 18 (*N*=4) and X (*N*=4). The 3p deletion region usually encompassed the whole-chromosome arm, although two cancers had smaller regions of change that involved the *BAP1* and *SETD2* loci but excluded *VHL* (see below). While deletions, and occasional copy-neutral LOH, of chromosome 3p usually involved all or most of the whole-chromosome arm, in a few cases, smaller changes occurred and these clearly targeted the region around the *SETD2* and *BAP1* loci (Fig. 2; Supplementary Table 4). One cancer, P17, had acquired two small regions of deletion, one around *SETD2* and the other around *BAP1*, although we did not detect pathogenic SNVs of either gene in this tumour (Supplementary Fig. 5).

We then searched for small (<1 Mb), focal SCNAs genome wide that might represent oncogene amplification or tumour suppressor deletion. After filtering for germline segmental duplications, 12 such regions were found (Supplementary Table 7), although none was recurrent. However, one of the focal SCNAs involved a gene with strong *a priori* importance in cancer, this being a deletion of ~1 Mb around *CDKN2A* in tumour P02.

In the eight cancers with whole-genome sequence data, we annotated and investigated the structural variants that were identified by the Complete Genomics pipeline based on the presence of split sequencing reads between or within chromosomes. Medians of 81 (range 65–564) intrachromosomal changes and 14 (range 7–127) interchromosomal changes were found per cancer. There was a strong correlation between the numbers of intra- and interchromosomal changes in each cancer (linear regression, *P*<0.00001). Although some known fragile site changes were detected (for example, *WWOX* and *FHIT*), no recurrent or clearly pathogenic fusion genes, intra-gene deletions or translocations were present (Supplementary Data 1). Specifically, none of the *TFE3* translocations previously reported in pRCC^32^ was detected. One outlying cancer (P09) had a very large number (*N*=691) of structural changes. Comparison with our SNP array data showed that this excess was accounted for by the changes on chromosomes 2, 4 and 6 that we had identified above as having chromothripsis-like events (Supplementary Fig. 3). On chromosome 2, the sequence data showed the gross rearrangements almost all to be intrachromosomal, resulting in segmental disorder and multiple copy number changes. By contrast, the rearrangements on chromosomes 4 and 6 were driven by reciprocal interchromosomal exchanges, suggesting chromoplexy (Supplementary Fig. 6).

A further large-scale mutation of interest was identified from a split read in the Complete Genomics data between chr1:27,080,702 (intron 16 of *ARID1A*) and chr1: 109,834,252 (close to *MYBPHL*) in cancer P13. This change was confirmed using the SNP array data to comprise a deletion from the short-arm telomere to a site within intron 4 of *ARID1A* (Supplementary Fig. 7). There was an accompanying structural change, predicted from split reads (Supplementary Data 1) to be a pericentric inversion, with the short-arm break point at the *ARID1A* intron 4 site and the long-arm break point at a small ~1.5 kb region of copy number gain close to *MYBPHL*. This change deletes the 5′ end of *ARID1A*, and is thus predicted to inactivate protein function.

### Single-sample clonality analysis

Clonal structure in all cancers was evaluated using Pyclone^33^ (Supplementary Fig. 8). Median predicted number of clones was 7 (range 1–22). Large-scale gains of chromosomes 7, 12, 16 and 17 almost always mapped onto the major clone. *BAP1* and *ARID2* mutations mostly mapped to major clones, whereas *SETD2* and Nrf2 pathway mutations were almost all predicted to be sub-clonal (Supplementary Table 8). Since the resolution of clonal prediction from single samples may be limited, especially where there are multiple rare sub-clones, we sought to validate these findings in multi-region sequencing (M-seq) studies.

### M-seq and intra-tumour heterogeneity

Papillary carcinomas of different stages were collected from four patients (RK30, RK36, GK101 and GK116; Table 1) and subjected to M-seq. For the multi-focal cancer GK116, 5 regions were analysed (regions 1–4 from GK116_1 and, for comparison, region 5 corresponding to GK116_2). Whole-exome sequencing to a median depth of 99 × (range 72–172 × ) was performed on DNA from each tumour region and from paired normal kidney (or for GK116, blood). The regional distribution of non-synonymous mutations was determined on the basis of ultra-deep Ion Torrent amplicon sequencing. On average, 20.6% (range 5–49%) of the validated non-synonymous SNVs were heterogeneous, that is, not detectable in all sampled regions of an individual tumour.

**Figure 3:**
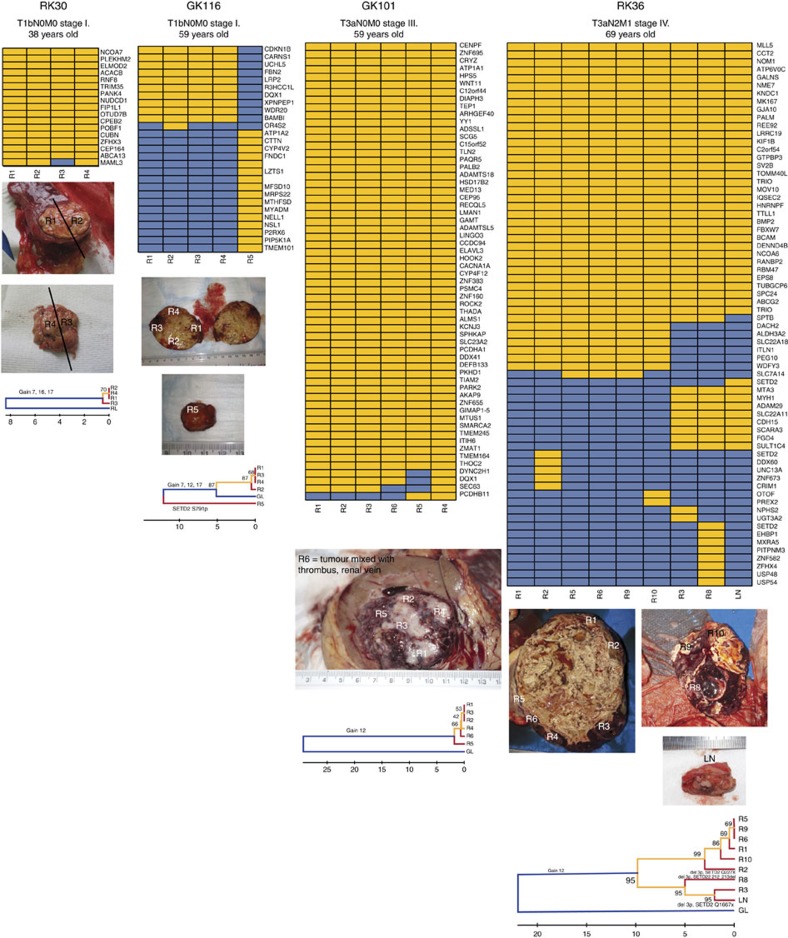
Regional distributions of non-synonymous somatic mutations in four pRCCs. For these M-seq cancers, the heat maps indicate the presence of a mutation (yellow) or its absence (blue) in each region. The non-M-seq sample GK116_2 is shown alongside GK116_1 for comparative purposes. Note that the *SETD2* mutation p.Glu1667X in RK36 LN was identified in the combined call of all regions of this tumour, but not called by the M-seq pipeline; subsequent inspection showed the M-seq call to be a false negative (Supplementary Fig. 11). Each picture shows the regions of core biopsies and regions harvested at nephrectomy. Phylogenetic trees were generated by UPGMA from Ion Torrent M-seq SNV data. Branch and trunk lengths are proportional to the number of non-synonymous mutations acquired. No cancer showed a significant difference between the spectra of SNVs present on the trunk or branches (*P*>0.05, details not shown). Putative driver SCNAs and SNVs are shown on their respective branch. For clarity, sub-clonal SCNA gains are not shown for the highly branched tumour RK36; these involve chromosomes 7, 16 and 17, and are present in regions 5, 9, 1, 10, 3, LN and (apart from chr17) 8. The apparent discordancy between the SNV-based trees and SCNAs may reflect chance, genomic instability, recurrent mutations or reversion mutations.

Somatic SNVs in the tumour regions were separated into mutations likely to be present in either the dominant or minority sub-clones, and we constructed phylogenetic trees by UPGMA (Unweighted Pair Group Method)^34^. The three earlier-stage tumours (RK30, GK116_1 and GK101) displayed minimal regional mutational heterogeneity or evidence of branched tumour evolution (Fig. 3), and the data were entirely consistent with the origins of GK116_1 and GK116_2 as separate cancers. The highest-stage cancer, RK36, displayed extensive branched evolution (Fig. 3). Although this patient was treated with sunitinib for 4 weeks before surgery, disease was stable during this period, and it is therefore unlikely that significant clonal selection occurred to account for the heterogeneity observed.

Similar diversity patterns were observed in regional copy number profiles (Supplementary Fig. 9), with relative SCNA uniformity found in the three earlier-stage tumours (Table 1), while RK36 exhibited greater intra-tumour heterogeneity. However, in contrast to somatic SNVs, ubiquitous and recurrent ‘driver’ events were identified—specifically, gains of chromosomes 7 and 17 in all regions of RK30, GK116_1 and RK36, and of chromosome 12 in GK101 and GK116 (Supplementary Fig. 9). However, RK36 showed some SCNA heterogeneity: while most regions (R1, R2, R3, R5, R6 and R10) showed haplotype-concordant gain of chromosomes 16 and 17, others (LN, R8 and R9) showed no copy number gain (Fig. 3; Supplementary Fig. 9). LN, R8 and R9 were not closely related by SNV analysis, suggesting that copy number losses subsequent to initial gains may have occurred independently in several regions. GK116_2 (region 5) had acquired gains of the same chromosomes as GK116_1 (regions 1–4). However, for chromosomes 7 and 17, the alternate haplotype was involved (Supplementary Fig. 10), consistent with independent tumour origins, but parallel evolution (that is, selection for the same oncogenic events in each pRCC from the same kidney).

We previously demonstrated parallel evolution in ccRCC involving multiple independent driver SNVs in different regions of the same cancer^34^. Here we identified parallel evolution in tumour RK36. This patient carried three spatially separated, truncating mutations in *SETD2* (p.Glu2277X in R2, p.Val212fs in R8 and p.Glu1667X in LN), all accompanied by 3p deletions that were not all concordant with respect to haplotype (Supplementary Fig. 10). These data support the role of *SETD2* as a sub-clonal driver in pRCC. Events such as this and the Nrf2 pathway mutations in the single-sample cancer RK133—converging on single genes in the same pathway—indicate remarkably strong selection for these mutations in particular tumours at certain stages of their growth.

## Discussion

Genome and exome sequencing have shown that pRCC driver mutations overlap with those of other cancers, but there are also genetic features specific to pRCC. We have identified probable pathogenic pRCC driver mutations in *BAP1*, *SETD2*, *ARID2* and the Nrf2 pathway genes, and where investigation by M-seq was possible, these mutations were sub-clonal drivers. This was evidenced most strikingly in the parallel evolution of tumour RK36 that acquired three distinct, truncating *SETD2* mutations associated with deletions of different 3p haplotypes in three regions out of nine tested. The fact that 7 of 31 pRCCs had no detectable SNVs or small insertion–deletions (indels) of probable pathogenic effect in the top genes (Table 2; Fig. 2) is also compatible with the presence of heterogeneous, sub-clonal driver SNVs that were not readily identifiable in those lesions. However, all but one of those seven cancers had acquired large SCNAs. This suggested that the major, truncal drivers for pRCC might be copy number changes. In keeping with this hypothesis, we typically found that large-scale copy number gains on chromosomes 7, 16 and 17 are often clonal changes that are strong candidates for major pRCC drivers, even though the genes targeted by these changes have not unambiguously been identified.

The pRCC driver genes identified by this study include *BAP1* and *SETD2*. Pathogenic *BAP1* and *SETD2* mutations have now been found in several cancer types^35^,^36^, especially in bladder tumours and ccRCC. Our additional focus on *ARID2* mutations as pRCC drivers was prompted by our initial finding that all three mutations in our discovery phase were protein truncating, and by the role that the ARID2 protein plays in chromatin remodelling. Interestingly, TCGA project has shown that *ARID2* is not significantly mutated in any individual cancer type studied to date, but is significantly mutated, although at a low frequency, in a pan-cancer analysis^37^. Our finding of somatic Nrf2 pathway (*KEAP1*, *CUL3* and NFE2L2) mutations in pRCCs is in line with a small, previous study^38^. It is possible that targeting Nrf2 signalling will have limited clinical potential given that the mutations are often sub-clonal, but there remains hope for such treatments given that one cancer acquired Nrf2 activation by both *KEAP1* and *NFE2L2* mutations in what appeared to be different cancer sub-clones.

We found very few clinicopathological–molecular associations in our samples (Table 1), but we did find differences between pRCC and ccRCC. It is established that pRCC differs markedly from ccRCC in the near-complete absence of *VHL* mutations in the former and their almost ubiquitous presence in the latter, and it appears from our study that pRCCs also have fewer *PBRM1* mutations than ccRCCs. However, these two genes lie on the same chromosome arm (3p) as *SETD2* and *BAP1*, which are mutated in both types of renal tumour. It has been proposed in ccRCC that, following a *VHL* mutation, one copy of 3p is deleted and that the other genes are ‘opportunistic’ in that they can act as tumour suppressors, while only requiring a single ‘hit’, because they have been rendered hemizygous by the *VHL* ‘second hit’ (summarized in ref. 39). The pRCC data show that a model whereby the other 3p mutations depend on preceding *VHL* mutations is unlikely to be correct, and that loss of 3p is an important event in pRCC development in the absence of *VHL* changes. However, it remains entirely possible that *SETD2* mutation can be opportunistic in both ccRCC and pRCC, depending on a preceding *BAP1* mutation and 3p deletion in the latter case. pRCCs also have relatively few mutations in ccRCC drivers such as *KDM5C*, *PTEN*, *MTOR* and *PIK3CA*, while ccRCCs have few *ARID2* mutations and gains of chromosomes 7, 16 and 17 are uncommon^40^. Mutations in the Nrf2 pathway genes appear to be specific to pRCC, but re-analysis of TCGA ccRCC data (details not shown) using Intogen does shows that a small, but significant proportion (~2%) has mutations in *NFE2L2* (ref. 18). Whether these tumours show any mixed ccRCC–pRCC features is unclear.

Our study has a few limitations. The different sequencing platforms used provided a potential problem, but there was no clear effect on SNV detection, which was the mainstay of our analysis. Indel detection did, however, show inter-platform differences and indel analysis had to be restricted to validated changes used in driver gene identification. Sample requirements also meant that SNP array data could not be obtained for the multi-region samples, and that complementary techniques, such as methylation and RNA sequencing, could not be used for any of the cancers. These limitations do not affect our findings or conclusions, but we cannot, for example, exclude a methylator pathway of tumorigenesis in some pRCCs. Finally, our sample size was sub-optimal for the detection of rare driver genes, providing a limited power to identify genes mutated in <15% pRCCs; this does not invalidate our positive findings, but means that the additional rare pRCC drivers may require projects involving many hundreds of cancers, at an even larger scale than the current TCGA analysis. Finally, additional clonal reconstruction and M-seq analyses will be required to pick apart the evolutionary complexity of pRCCs and to confirm or refute our model that copy number gains typically drive the initial stages of pRCC pathogenesis, with many driver SNVs acting in sub-clones.

In conclusion, sequencing of papillary renal cancer exomes and genomes has identified similarities to and differences from the more common clear-cell type of kidney cancer. Shared mutated genes include *BAP1* and *SETD2*, but mutations in genes such as *ARID2* and *KEAP1* are specifically associated with pRCC. In general, our model is that the evolution of pRCCs proceeds from truncal, chromosome-scale copy number changes, such as that involving chromosome 7, to the acquisition of sub-clonal-specific driver mutations. Although *VHL* and *PBRM1* mutations are known to be frequent truncal drivers in ccRCCs^9^, similar high-frequency, major pRCC driver mutations appear not to exist in pRCCs. The predominance of large, truncal SCNAs in pRCC highlights an arguably under-recognized mode of tumorigenesis, and therapeutic targeting of these SCNAs is likely to be problematic, given the number of potential driver events present within each region.

## Methods

### Sample description

The discovery set comprised 31 papillary renal cell cancers, paired with peripheral blood or normal tissue that had been collected at nephrectomy from unrelated individuals from the United Kingdom. Ethics approval was obtained from Oxfordshire Research Ethics Committee C (project 09/H0606/5) for analysis of anonymized samples for driver gene discovery. Samples for M-seq were collected from individuals enrolled in the ‘Response and Resistance to Targeted Therapy in Renal Cell Carcinoma’ tissue collection protocol of the London Renal Cancer Consortium (ethics approval reference 11/LO/1996) and informed consent was obtained from participants. Tumour morphology was reported after collection by the local histopathologist and then reviewed by another histopathologist (S.F.). This confirmed pRCC in all cases (Table 1), with >70% cancer cells in the tumour specimens. Genomic DNA was extracted from each tumour and paired blood or normal tissue and quantified using standard methods.

The replication set comprised 60 frozen or formalin-fixed, paraffin-embedded (FFPE) pRCCs. Each sample had >60% cancer cells, and was subject to enrichment for malignant cells using haematoxylin and eosin-stained-sections as a guide. After homogenization where necessary, DNA was extracted using the Qiagen Dneasy Blood and tissue kit according to the manufacturer’s instructions.

### Multi-region sampling

We isolated between 4 and 9 samples of 10 × 5 × 5 mm, representing the spatial extent of the primary tumour, from each nephrectomy specimen. Samples were macrodissected to minimize the stromal contamination, and half of each sample was snap frozen in liquid nitrogen within 1 h of clamping the renal artery. Sample collection was performed according to strict standard operating procedures in all cases and included photographic documentation. DNA and RNA were extracted using the Qiagen AllPrep micro kit following the manufacturer’s instructions. Nucleic acid yields were determined by Quibit (Invitrogen). Regions which histopathological review judged to contain <70% tumour cells were excluded.

### Single-sample sequencing

Paired-end sequencing of eight tumour and paired normal samples were performed using the Complete Genomics (Mountain View, California) service. The Complete Genomics’ CGAtools package was used to map reads to the hg19 reference genome and call variants, including somatic mutations, in the tumour-normal pairs. Standard Complete Genomics quality filters were used. A list of potential structural variants was also provided by CGAtools. Variants were annotated with ANNOVAR^41^ using hg19 reference genome and 2013 versions of standard databases and functional prediction programs. We excluded duplicated genomic regions (>90% homology) from the analysis and variants within regions with low mappability scores (≥3 locations per genome). Variants were annotated with ANNOVAR (RefSeq gene models) using: dbSNP (132); 1,000 genomes project allele frequencies (November 2011); University of California Santa Cruz (UCSC) segmental duplication scores; and UCSC 46 species conservation scores; and predictions of functional importance from SIFT and PolyPhen2.

Exome capture was performed using the Agilent SureSelect (*N*=7) or Illumina TruSeq (*N*=8) kits. Samples were quantified using the Qubit system (Invitrogen) and sequencing libraries constructed from 1 μg DNA post capture using the NEBNext DNA Sample Prep Master Mix Set 1 Kit (NEB). Ligation of adaptors was performed using 6 μl of the Illumina Multiplexing Sample Preparation Oliogonucleotide Kit. Libraries were size-selected using 2% gel electrophoresis and the distribution of fragments in the purified fraction was determined using the Tapestation 1DK system (Agilent/Lab901). Each library was PCR-enriched using the following custom primers:

Multiplex PCR primer 1.0: 5′-AATGATACGGCGACCACCGAGATCTACACTCTTTCCCTACACGACGCTCTTCCGATCT-3′

Index primer: 5′-CAAGCAGAAGACGGCATACGAGAT[INDEX]CAGTGACTGGAGTTCA GACGTGTGCTCTTCCGATCT-3′

Indexes were 8-bp long and part of an indexing system developed in-house. Four independent PCR reactions per sample were prepared using 25% volume of the pre-PCR library each. After eight cycles of PCR (cycling conditions as per Illumina recommendations), the four reactions were pooled and purified with AmpureXp beads. The final size distribution was determined using the Tapestation 1DK system (Agilent/Lab901). The concentration of each library was determined by the Agilent qPCR Library Quantification kit. Samples were sequenced using the Illumina HiSeq2,000 platform as paired 100-bp reads with Chemistry version 3.0, with the aim of a target average coverage of 100 × for the blood DNA and 200 × for the tumours. After removal of PCR duplicates using Picard, reads were mapped with Stampy version 1.0.12 (r975) 18 onto the Human Reference Genome to (GRCh37d5/hg19). SNVs and small indels were called with Platypus version 0.2 using the tumour-normal pairs of bam files together to ensure comparable calls at every locus. Variants were only called if they were assigned a sufficiently high posterior probability (phred score of 5). We removed the allele bias filter to increase sensitivity. Finally, for selected variants, we made sure the automatic call matched the data by expert visual inspection of the mapped reads onto the reference genome using read direction colouring on top of the standard integrated genomics viewer (IGV) scheme (http://www.broadinstitute.org/igv/alignmentdata/). We had previously used Ion Torrent technology to show that the Illumina-Stampy-Playtpus pipeline produces >95% validated variants in comparable cancer samples^42^. Annotation was performed as for the Complete Genomics samples.

For filtering of variant calls for analysis, calls were first compared between matched constitutional and tumour samples to identify somatic mutations. For analysis of mutation burden and spectra, we applied the following exclusion filters to somatic variants: (i) presence in a segmental duplication region or a region with mappability score <0.5; (ii) variant present in any read from the paired normal sample; (iii) fewer than 10 reads in total at the variant site in the normal sample; (iv) fewer than eight reads in total in the tumour; (v) fewer than three variant reads in the tumour; (vi) variant allele frequency <10% in the tumour; and (vi) presence of variant in public databases (Exome Variant Server, 1,000 genomes project, Complete Genomics 69 reference genomes) at a frequency of >1%. Variants identified in constitutional DNA from any of the other local, non-cancer sequencing projects (for example, 29 million variants across 284 samples from the Oxford-Illumina WGS500 consortium) were discarded as being more likely due to systematic error in our pipeline than genuine somatic mutation. For driver gene identification, we enriched further for high-confidence calls. We therefore applied the following exclusion filters to somatic variants: (i) presence in a segmental duplication region or a region with mappability score <0.5; (ii) frequency in 500 locally sequenced constitutional genomes of >%1, or Exome Variant Server >1%, 1,000 genomes project >1% or Complete Genomics 69 reference genomes >10%; or (iii) variant read depth below 10 × .

### Targeted Sanger sequencing

Validation in the original discovery set and replication in the set of 60 additional pRCCs were performed for mutations in *BAP1*, *SETD2* and *ARID2*, and validation of *FH*, *MET* and *KEAP1* using bidirectional Sanger sequencing of the coding regions of each gene (details available from authors). For *SETD2*, analysis was restricted to the SET, WW and SRI domains of the protein that show a degree of mutation clustering. Any variants found were replication-tested in a second, independent DNA sample from the same tumour. The somatic origin of any variation from the human reference sequence was confirmed by analysis of paired DNA from blood or normal tissue. Mutations were analysed with Mutation Surveyor V3.97 and confirmed by inspection of electropherograms.

### Ion Torrent validation sequencing

As previously employed^42^, a custom 75 cancer-gene exome panel (details available on request) was used for technical validation of sequencing in the non-M-Seq cancers. Approximately 100 ng DNA was used for sequencing. Mean read depth was 1,067. Variants present in the genome and exome sequence data were assessed alongside the equivalent Ion Torrent data, using both automatic calls in the Torrent Server output and visual inspection using the IGV.

### SNP arrays

Twenty-three tumours were genotyped using the Affymetrix 2.7 M Cytogenetics array or the CytoScan HD array. The data were analysed using Chromosome Analysis Suite (ChAS) 2.1. Copy number changes and LOH were called using the software high-resolution settings, which allowed detection of ‘mosaic’ changes. Copy number variants that overlapped by >50% with germline copy number variants were filtered out.

### Clonal structure of single-sample tumours

Allelic heterogeneity, and thus clonal structure, was evaluated from whole-cancer samples using an in-house program based on PyClone version 12.3.1. Allelic frequencies of selected somatic mutations were obtained using the number of reads and the number of reads carrying a variant as the total copy number. The copy number value at each of these loci and the tumour content were obtained from the ChAS program as described above. For each sample, all exome-wide somatic mutations were used to characterize heterogeneity. Selected copy number changes (gains of large regions of chromosomes 7, 16 and 17) were also included in the Pyclone analysis using dummy composite number of reads and number of reads carrying a variant values for all somatic variants within regions defined using ChAS.

### Mutation significance analysis

Gene-based and pathway analyses to detect significantly over-represented mutant genes and pathways were performed by Intogen 23 and MutSigCV using the annotated, quality-filtered, somatic mutations from all cancers.

### M-seq and cancer evolution analysis

Exome capture was performed on 3 μg of genomic DNA per sample with the Agilent SureSelect Human All Exon V4 kit according to the manufacturer’s instructions, and paired-end multiplex sequencing of samples on the Illumina Genome Analyzer II and HiSeq platforms at Cancer Research UK London Research Institute (LRI; RK30, RK36, GK101 and GK116_1). Genomic DNA sequenced at LRI was randomly fragmented by Covaris to obtain fragments distributed between 250 and 300 bp in length. Adaptors were ligated to both ends of the fragments and adaptor-liagted templates were purified using Agencourt AMPure SPRI beads. Extracted DNA was amplified by ligation-mediated PCR, purified and hybridized to the Sure Selected biotinylated RNA library (BAITS) for enrinchment. Each capture library was loaded on the Ilumina platform, and paired-end sequencing was performed to the desired median sequencing depth (~60 × ).

To provide a consistent comparison with our previous data on ccRCC^9^, we used the following special pipeline to analyse the M-seq sequencing output. Reads were aligned to hg19 using BWA 0.5.9 (r16) with a seed length of 100 and up to 4 mismatches allowed. Duplicate reads were removed using Samtools before analysis. Single-nucleotide variant calling was performed using CAVEMAN^9^,^43^ and small insertions and deletions were identified using a modified version on Pindel in paired tumour-normal mode. The following filtering criteria were applied to the called variants: only nucleotides with Phred quality of 20 or greater were considered; only reads mapping uniquely to the genome were considered; a minimum of 10 × coverage in both germ line and tumour was required; a minimum of two instances of the variant in the tumour region were required; and variants in positions listed in dbSNP 132 were removed. Somatic mutations present in at least 5% of the reads based on exome sequencing in at least one tumour region were further analysed. To increase specificity, only simple insertions and deletions events of <10 bp were selected. In-house filter software was used to extract high-quality indels: considering the high-sequence coverage obtained in these samples, only those indels with a minimum coverage of 20 reads in both tumour and normal samples and with a minimum frequency of 10% of the reads and also a minimum of 5 independent reads supporting the event on the tumour sample and with no evidence in the normal sample were considered.

Putative variants of interest were manually inspected in IGV before validation by Ion Torrent sequencing. Similar to Illumina-Stampy-Platypus, we had shown the Illumina-BWA-CAVEMAN pipeline previously generated a >90% Ion Torrent validation rate^9^. We created custom Ampliseq (Lifetech) validation panels for non-synonymous somatic mutations and indels called in at least one region using the AmpliSeq Designer (http://www.ampliseq.com/). Multiplex PCRs were performed according to the manufacturer’s instructions with the tumour-specific primer pool on DNA from each region of the tumours. Amplicon pools were used for the construction of barcode sequencing libraries and these were multiplex sequenced on the Ion Torrent PGM sequencer, to a mean target depth of 500 × (Lifetech). A mutation was considered to be present in a tumour region if a non-synonymous mutation or indel was detected in at least 1% of the ultra-deep reads, thresholds selected on the basis of the error rate of the sequencing platform. Mutations that passed validation were included in the phylogenetic analysis.

For ploidy profiling, a suspension of nuclei was created from fresh tumour tissue and washed with PBS, and then fixed with 70% ethanol. After 60 min, nuclei were washed again with PBS and stained with propidium iodide. Flow cytometric analysis of DNA content was performed using the BD LSRFortessa cell analyser, BDFacsDiva software and FlowJo software. The DNA index of an aneuploid peak, where present, was calculated by dividing G1 peak of the aneuploid population by G1 peak of the normal diploid cells.

For copy number analysis, relative copy number was estimated from whole-exome-sequencing data using VarScan2 (v2.2.11)^44^ with default parameters, excluding the sex chromosomes and low mapability regions (ENCODE 'DAC blacklisted' regions) and adjusting for GC content. To identify genomic segments of constant copy number, logR values were quantile normalized, winsorized using the median absolute deviation, and jointly segmented at the patient level (gamma=1,000). Absolute (integer) copy numbers were derived from relative copy numbers using ABSOLUTE (v1.0.6)^45^. SNVs with ≥50 × sequencing coverage were included in the analysis and AmpliSeq-derived variant allele frequencies were used where possible. Minimum/maximum ploidy was set to within ±0.5 of the prior ploidy estimate, calculated from the sample’s fluorescence-activated cell sorting-based DNA index. Subsequently, the top five ABSOLUTE models (ranked by log likelihood) were retrieved for each exome, and a set of inter-sample models was identified that minimized the total pairwise distance derived from the segments’ expected modal copy number, while maximizing the model’s posterior log likelihood. Final model solutions were manually reviewed as recommended. Finally, adjacent segments of equal clonality and absolute copy number were merged. To compare the haplotypic origin of shared SCNAs between tumour regions, allele frequencies for each heterozygous SNP were estimated. SNP alleles were classed as ‘major’ (allele frequency>0.5) or ‘minor’ (allele frequency<0.5) in the highest cellularity region (as defined by ABSOLUTE) of each tumour, assuming that the ‘major’ allele represents the higher-copy number haplotype where applicable. Patterns of major/minor allele frequency were then compared with these reference regions; a SCNA with an inverted major/minor allele frequency distribution was interpreted as a recurrent (or secondary) SCNA affecting the alternate haplotype.

### Phylogenetic analysis of multi-region cancers

Phylogenetic relationships between tumour regions were inferred using the UPGMA as implemented in MEGA6 (ref. 46). The evolutionary distances were calculated using the number of differences between regions, and uncertainty assessed by a bootstrap test (1,000 replicates). Trees are shown drawn to scale, with branch lengths in the same units as those of the evolutionary distances used to infer the phylogenetic tree, and the percentage of replicate trees in which regions clustered together in the bootstrap test shown next to the branches.

## Author contributions

Mi.K., C.N., S.H., M.S., F.C.-G., Mo.K., I.V., C.S. and I.T. analysed the sequencing data. C.N., C.B., A.Ro., E.deB., A.J., K.Ho., R.F., E.J., N.M., B.P., S.B. and A.Ra. performed the laboratory experiments. R.F., J.W., M.G., J.L., M.S., L.P., M.G., D.L.N., S.H., T.O.B., B.C., A.C., C.H., A.Po., R.B., L.T., S.E.-C., A.Pr., E.M., M.E.B. and S.F. recruited patients and provided samples. G.S., M.E.B., A.C., C.H., A.Po. and S.F. performed histological analysis. P.R., K.He., C.S. and I.T. oversaw and planned the study. Mi.K., C.N., S.H., M.S., C.B., C.S. and I.T. wrote the manuscript.

## Additional information

**Accession codes:** Exome and genome sequence data for papillary renal cell carcinoma samples have been deposited in the European Nucleotide Archive under the primary and secondary accession numbers PRJEB7875 and ERP008861.

**How to cite this article:** Kovac, M. *et al.* Recurrent chromosomal gains and heterogeneous driver mutations characterise papillary renal cancer evolution. *Nat. Commun.* 6:6336 doi: 10.1038/ncomms7336 (2015).

## Supplementary Material

Supplementary Figures, Tables, Methods and References Supplementary Figures 1-11, Supplementary Tables 1-8, Supplementary Methods and Supplementary References

Supplementary Dataset 1 Putative structural somatic mutations identified on the basis of split reads in pRCCs P08-15 with whole-genome sequence data

## Figures and Tables

**Table 1:** Clinicopathological data and summary mutation data for each pRCC.

**ID**	Age	Sex	Stage	Type	Grade	Sequencingmodality	M-seq	Exomecoverage >30 ×	SNV no.	dN/dS	Ts:Tv	Chromosomalgains	Chromosomaldeletions	Totalchromosomalchanges
P01	38	M	T1bN2Mx	1/2/CDC	3	1		94.0%	45	1.72	0.78	1q, 2p, 17q	3p, 6q, 11q, 13q, 14q, 21q	9
P02	85	F	T3aN2MX	2	3	1		93.5%	57	2.36	0.82	1q, 2, 6p, 7, 12p, 13q, 16q, 17q, 18p, 19q	3p, 17p, 18q	12
P03	86	F	T1bNXMX	2	2	1		93.3%	44	1.98	1.08	7, 8, 16, 17, 20	X	6
P04	55	M	T3bN2MX	1/2	3	1		82.4%	55	1.75	1.89	—	—	0
P05	57	M	T1aNXMX	2	2	1		93.3%	2	0.50	1.00	7, 16, 17, 21q	9p, X	6
P06	62	M	T1aN0M0	2	3	1		94.9%	87	2.67	0.58	3q, 7, 12, 16, 17	X	6
P07	38	F	T1aN0M0	2	2	1		93.8%	185	1.87	0.24	—	—	0
P08	72	M	T3aNXMX	1/2	3	2		83.1%	30	3.00	1.69	7, 16p	—	2
P09	75	M	T1aN0MX	2	3	2		78.7%	79	3.95	1.06	2q	1p, 2p, 2q, 3p, 3q, 4q, 6p, 7q, 9, 18, 20p	12
P10	63	F	T2N0MX	1/2	3	2		82.8%	77	2.19	0.94	2, 3, 5, 7, 12, 16, 17	—	7
P11	30	F	T1aN0MX	2	3	2		81.7%	63	3.68	1.32	—	14q, 21q, 22q	3
P12	44	F	T2NXMX	2	3	2		84.3%	46	1.28	2.15	—	—	0
P13	76	M	T2NXMX	2	3	2		78.2%	94	2.56	0.89	12, 16, 17	—	3
P14	62	M	T2NXMX	2	3	2		85.6%	71	1.98	0.97	7, 16, 17, 20	X	5
P15	57	F	T1aNXMX	2	3	2		77.3%	83	2.56	0.66	16, 17q	—	2
P16	48	M	T1aNXMX	2	1	3		90.5%	42	3.46	1.27	7, 16, 17	—	3
P17	44	F		2	3	3		91.2%	243	1.68	1.77	2q, 5, 7, 12, 16, 17	3p	7
P18	46	F	T1NXMX	2	2	3		89.1%	64	4.22	1.04	—	—	0
P19	60	M	T1NXMX	2	2	3		85.7%	5	0.63	1.12	7	—	1
P20	63	M	T1aNXMX	2	3	3		87.3%	77	3.20	0.72	17	—	1
P21	87	F	T2NXMX	2	2	3		89.9%	100	2.46	0.71	3, 8, 12, 16	3p, 9, 11, 18, 22	9
P22	76	M	T3bNXMX	1/2	2	3		84.9%	75	2.90	0.95	7, 12, 17, 20	3, 18	5
P23	80	M	T3bN0MX	2	3	3		90.0%	95	3.32	0.90	1q, 9, 16p, 17q	2p, 2q	6
RK30	38	F	T1bN0M0	1	2	3	Yes	90.3%	199	3.13	0.21	7, 16, 17	1p, 19, 22	6
RK36	69	F	T3aN2M1	1	3	3	Yes	85.9%	23	1.67	0.37	Complex	Complex	Complex
GK101	59	M	T3aN0M0	2	4	3	Yes	89.6%	90	3.51	0.67	12	9, 14, 22	4
GK102	20	F	T1bN0M0	1		3		84.6%	132	1.62	1.59	Complex	Complex	Complex
GK116_1	59	M	T1bN0M0	1		3	Yes	93.7%	58	5.92	1.02	7, 12, 17	22	4
GK116_2	59	M	T1bN0M0	1		3		92.0%	72	3.35	1.49	2, 3, 7, 12, 16, 17	21	7
GK116_3	59	M	T1bN0M0	1		3		92.4%	55	1.57	1.25	7, 12, 17	18	4
RK133	68	M	T2aN0M0	2	2	3		90.0%	69	2.50	0.59	7, 16, 17		3

CDC, collecting duct cancer features; F, female; grade, Fuhrman grade; ID, cancer ID; M, male; pRCC, papillary renal cell carcinoma; SNVs, single-nucleotide variants.

Basic demographic, clinical and histopathological data are shown. Age=age at presentation. . Stage denotes TNM classification. Type denotes morphological type 1 or 2; cancers with mixed features or disagreement between pathologists are shown with ‘+’. The sequencing modality is: 1, Agilent or Illumina exon capture with sequencing in Oxford; 2, Complete Genomics whole-genome sequencing; 3, Illumina exon capture and sequencing in London. M-seq denotes a tumour for which multi-region sequencing was performed; in subsequent data, for the multi-region cancers, only the region with the highest mutation burden is displayed to provide a valid comparison with the other tumours. Coverage >30 × =proportion of the exome covered at >30 × read depth. SNV number denotes number of high-quality, exonic somatic SNVs. dN/dS denotes ratio of non-synonymous to synonymous somatic exonic mutations. Ts:Tv denotes ratio of somatic exonic transitions to transversions. Chromosomal gains and Chromosomal deletions denote large chromosomal SCNAs involving >30% of any chromosome arm from SNP array data. For completeness, similar large SCNAs from exome sequencing are also shown for the eight cancers without SNP array data, but only if a whole-chromosome arm was involved. For two cancers, RK36 and GK102, a very complex picture was found, perhaps owing to polyclonality. Total number of chromosomal changes is also shown. ‘—’ denotes not found. Blank cells denote data not obtained. Further data are in Supplementary Fig. 4.

**Table 2:** Putative somatic driver mutations.

**Chr**	Start	Ref	Alt	Mutation type	Sample ID	Gene	DNA change	Protein change
2	178,098,815	T	G	Non-synonymous SNV	P20	NFE2L2	c.230A>C	p.D77A
2	178,098,957	G	A	Non-synonymous SNV	RK133	NFE2L2	c.C40T	p.L30F
2	225,368,499	AT	—	Frameshift deletion	P16	CUL3	c.1246_1247del	p.416_416del
3	47,098,445	G	A	Stopgain SNV	RK36	SETD2	c.C6829T	p.Q2277X
3	47,125,708	C	del52bp	Frameshift deletion	P21	SETD2	c.5562insCAAGCCdel58bp	p.P1854fs
3	47,142,964	G	A	Stopgain SNV	RK36	SETD2	c.C4999T	p.Q1667X
3	47,163,755	A	G	Non-synonymous SNV	GK116_2	SETD2	c.T2371C	p.S791P
3	47,165,490	GGCC	—	Frameshift deletion	RK36	SETD2	c.636_639delGGCC	p.V212fs
3	52,437,911	C	T	Splicing	GK102	BAP1	c.1251-1G>A	splicing
3	52,441,217	C	T	Non-synonymous SNV	P21	BAP1	c.553G>A	p.G185R
3	52,442,082	G	—	Frameshift deletion	P02	BAP1	c.267delC	p.N89fs
5	14,368,959	G	A	Non-synonymous SNV	RK36	TRIO	c.G3017A	p.R1006H
5	14,401,132	G	A	Nons-ynonymous SNV	P18	TRIO	c.4675G>A	p.V1559M
5	14,488,232	T	C	Non-synonymous SNV	RK36	TRIO	c.T7495C	p.S2499P
5	14,498,309	G	A	Non-synonymous SNV	GK116_3	TRIO	c.G8159A	p.G2720D
7	4,839,909	—	G	Frameshift insertion	P15	RADIL	c.2875_2876insC	p.P959fs
7	4,855,034	C	A	Non-synonymous SNV	P11	RADIL	c.2014G>T	p.A672S
7	4,855,997	G	A	Non-synonymous SNV	P20	RADIL	c.1828C>T	p.R610C
7	91,630,828	C	G	Non-synonymous SNV	GK101	AKAP9	c.C1597G	p.L533V
7	91,631,082	—	T	Frameshift insertion	P12	AKAP9	c.1851_1852insT	p.S617fs
7	91,712,923	A	G	Non-synonymous SNV	P17	AKAP9	c.8576A>G	p.E2859G
7	116,339,937	G	A	Non-synonymous SNV	RK36	MET	c.G799A	p.E267K
8	144,990,455	C	T	Non-synonymous SNV	P11	PLEC	c.13438G>A	p.G4480S
8	144,992,402	G	A	Non-synonymous SNV	P07	PLEC	c.11491C>T	p.R3831W
8	145,000,007	C	A	Stopgain SNV	P10	PLEC	c.3994G>T	p.E1332X
8	145,006,606	G	A	Non-synonymous SNV	P13	PLEC	c.1843C>T	p.R615C
10	17,089,584	—	A	Frameshift insertion	P10	CUBN	c.3158_3159insT	p.T1053fs
10	17,110,636	C	T	Non-synonymous SNV	RK30	CUBN	c.G2759A	p.G920D
10	17,113,514	T	C	Non-synonymous SNV	P23	CUBN	c.2536A>G	p.I846V
10	17,165,680	G	T	Non-synonymous SNV	RK30	CUBN	c.C396A	p.D132E
12	46,243,511	—	A	Frameshift insertion	P11	ARID2	c.1864_1865insA	p.V622fs
12	46,244,150	TA	GTAC	Frameshift substitution	P15	ARID2	c.2244_2245GTAC	p.S748fs
12	46,287,469	A	—	Frameshift deletion	P01	ARID2	c.5328delA	p.L1776fs
13	103,388,350	C	A	Non-synonymous SNV	P03	CCDC168	c.14697G>T	p.R4899S
13	103,395,330	C	G	Non-synonymous SNV	GK102	CCDC168	c.G7717C	p.E2573Q
13	103,395,332	A	—	Frameshift deletion	GK102	CCDC168	c.7715delT	p.I2572fs
13	103,395,338	A	T	Non-synonymous SNV	GK102	CCDC168	c.T7709A	p.V2570E
13	103,400,929	TT	—	Frameshift deletion	P10	CCDC168	c.2117_2118del	p.706_706del
16	58,554,883	CAT	—	Non-frameshift deletion	P06	CNOT1	c.7108_7110del	p.2370_2370del
16	58,577,328	—	A	Frameshift insertion	P11	CNOT1	c.4617_4618insT	p.C1539fs
16	58,610,470	A	—	Frameshift deletion	P10	CNOT1	c.1601delT	p.I534fs
17	17,117,141	—	C	Frameshift insertion	P15	FLCN	c.1568_1569insG	p.K523fs
17	57,093,106	ATCAA	—	Frameshift deletion	P16	TRIM37	c.2437_2441del	p.813_814del
17	57,134,346	A	—	Frameshift deletion	P02	TRIM37	c.1089delT	p.F363fs
17	57,181,680	—	T	Frameshift insertion	P08	TRIM37	c.97_98insA	p.K33fs
17	60,028,290	A	C	Non-synonymous SNV	GK101	MED13	c.T6187G	p.L2063V
17	60,062,442	—	T	Frameshift insertion	P10	MED13	c.2395_2396insA	p.K799fs
17	60,072,560	—	T	Frameshift insertion	P11	MED13	c.2134_2135insA	p.K712fs
19	10,597,406	C	—	Frameshift deletion	P13	KEAP1	c.1797delG	p.S599fs
19	10,602,620	G	C	Non-synonymous SNV	RK133	KEAP1	c.C958G	p.R320G

pRCCs, papillary renal cell carcinomas; SNVs, single-nucleotide variants.

Data are from the discovery set of 31 pRCCs with exome or genome sequence data. The table shows genes in which three or more cancers carried somatic mutations of moderate or greater predicted functional effect. In addition, selected somatic mutations in genes of known or potential importance in renal cancer are shown. Reference accession numbers are: *NFE2L2* NM_006164, *CUL3* NM_003590, *SETD2* NM_014159, *BAP1* NM_004656, *TRIO* NM_007118, *RADIL* NM_018059, *AKAP9* NM_147185, *MET* NM_000245, *PLEC* NM_201381, *CUBN* NM_001081, *ARID2* NM_152641, *CCDC168* NM_001146197, *CNOT1* NM_016284, *FLCN* NM_144997, *TRIM37* NM_015294, *MED13* NM_005121 and *KEAP1* NM_012289.
