# Supplementary material for: Recurrent chromosomal gains and heterogeneous driver mutations characterise papillary renal cancer evolution
Source: Nat Commun. 2015 Mar 19;6:6336. doi: 10.1038/ncomms7336 (PMC4383019; doi:10.1038/ncomms7336)
Supplement: Supplementary Figures, Tables, Methods and References — Supplementary Figures 1-11, Supplementary Tables 1-8, Supplementary Methods and Supplementary References [file ncomms7336-s1.pdf]

Supplementary Information

Supplementary Figures

Supplementary Figure 1. The proportion of somatic SNVs in each tumour is shown in a trinucleotide context. See file “Supp_Data_1_Somatic_SNV_spectra_in_each_tumour”. Note that the mutation burden for some cancers (P05, P19) was very small.

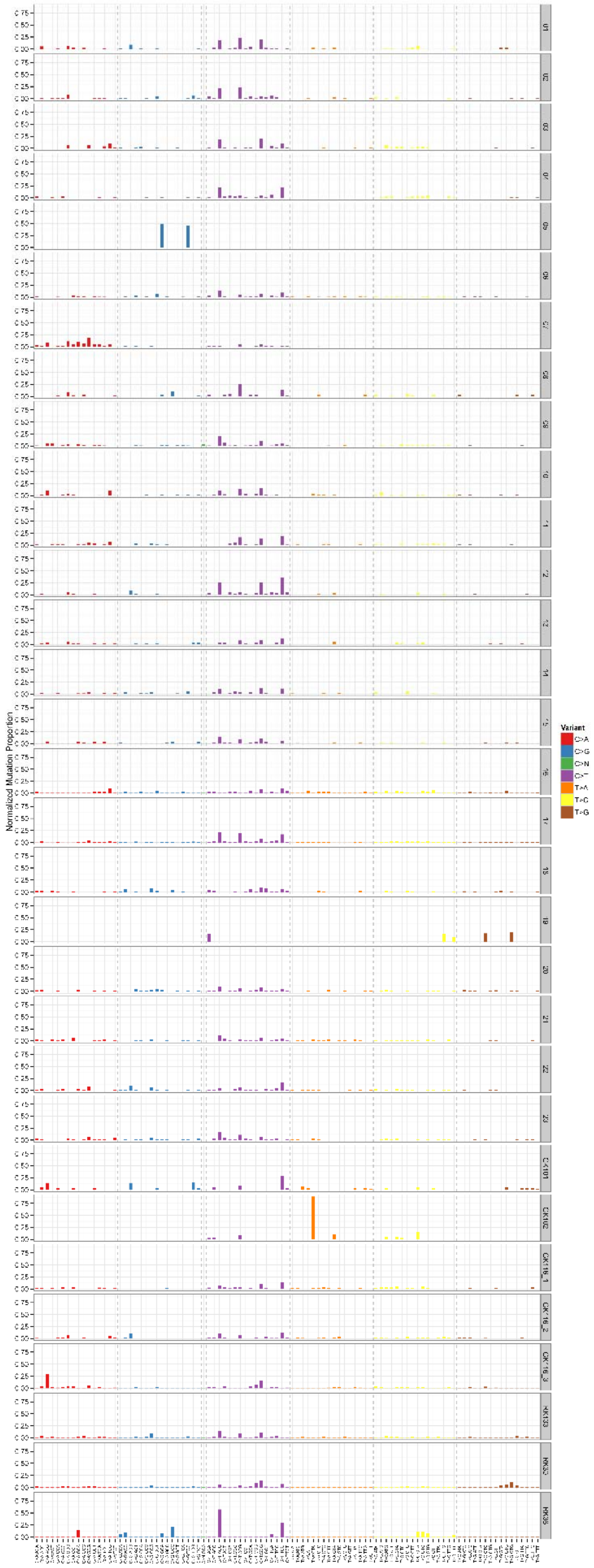

Supplementary Figure 2. SNCA burden density plot showing possible bimodality.

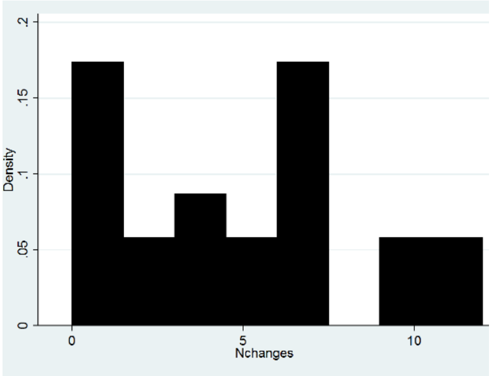

Supplementary Figure 3. Regions of chaotic chromosome rearrangement in tumours P09 (upper) and P23 (lower).

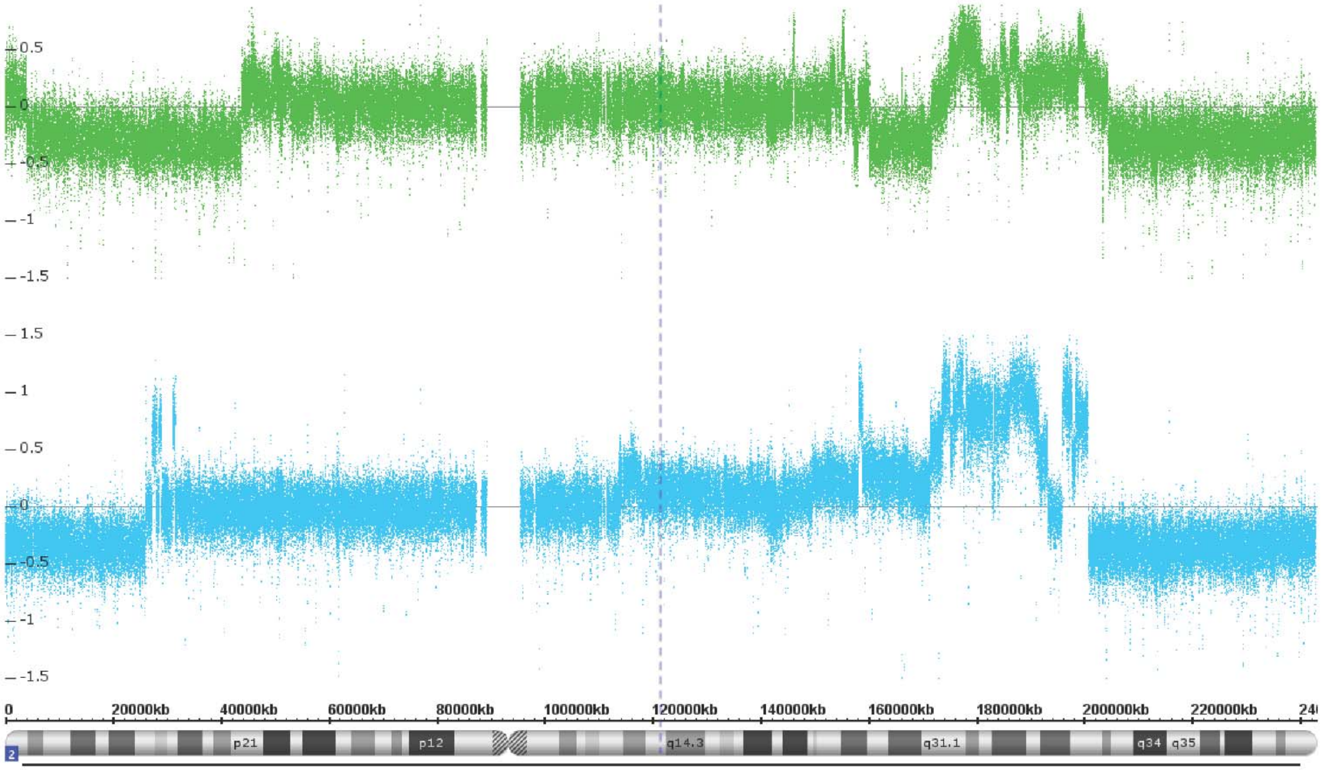

The plots show copy number along chromosome 2 in these tumours, perhaps resulting from chromothripsis. Note the similarities between the pattern of changes in each cancer, suggesting intrinsic fragility and/or strong selection. Whether any of these events, or the similar events on chromosomes 4 and 6 in P09 had any functional consequence remains unclear.

Supplementary Figure 4. Autosomal SCNAs, (i) overall, (ii) in cancers with SNP array data and (iii) in cancers without SNP array data.

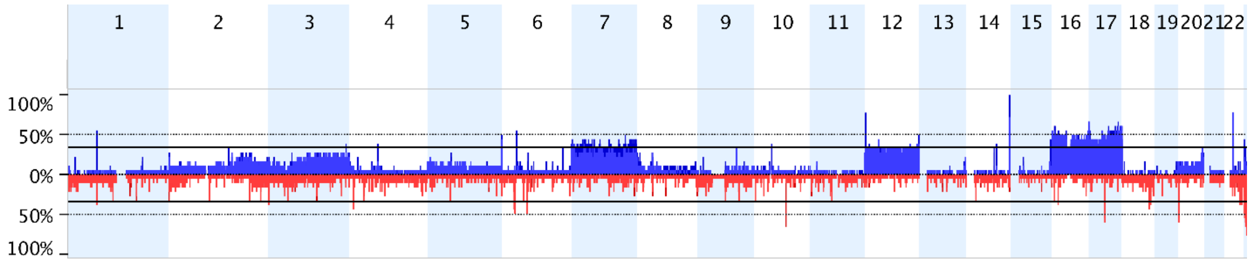

(i) Overall SCNA frequency in 23 cancers with SNP array data. Blue=gain, red=deletion

P01

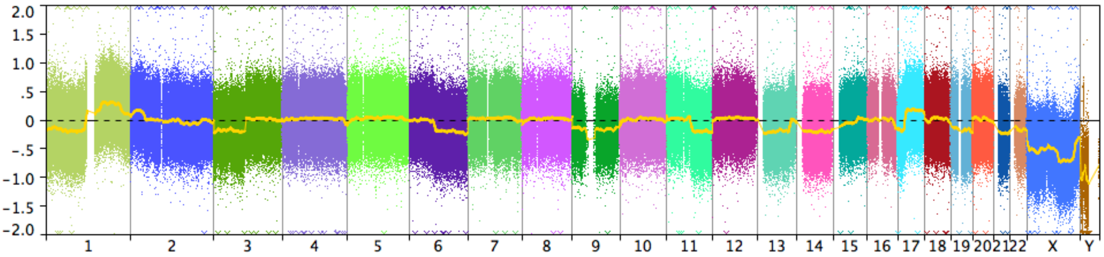

P02

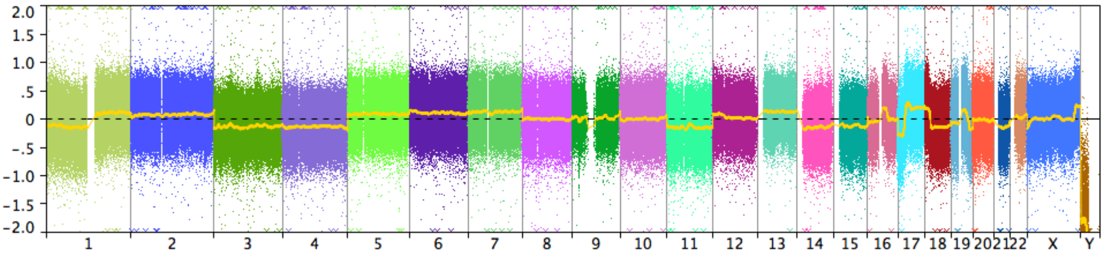

P03

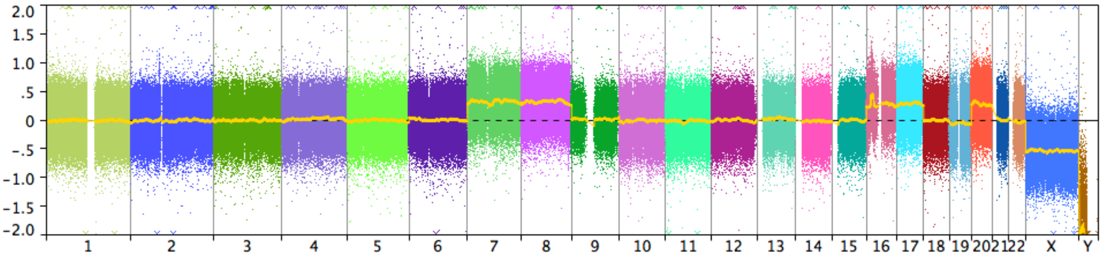

P04

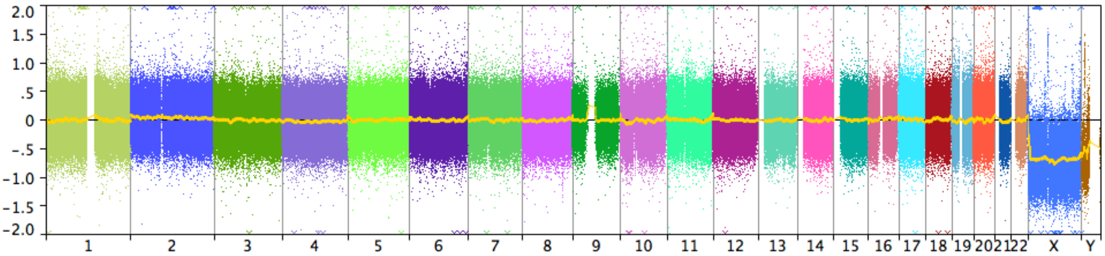

P05

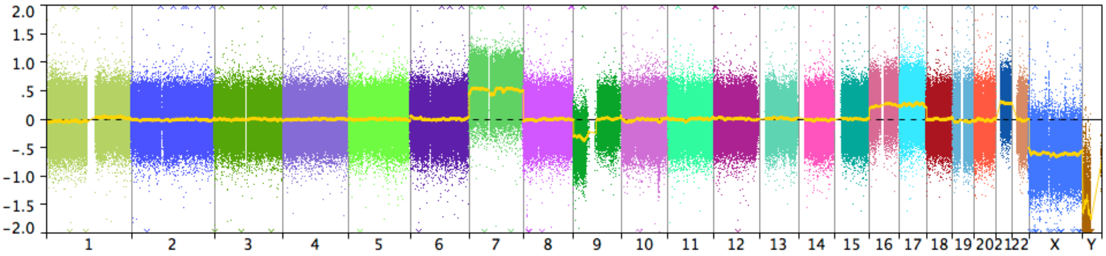

P06

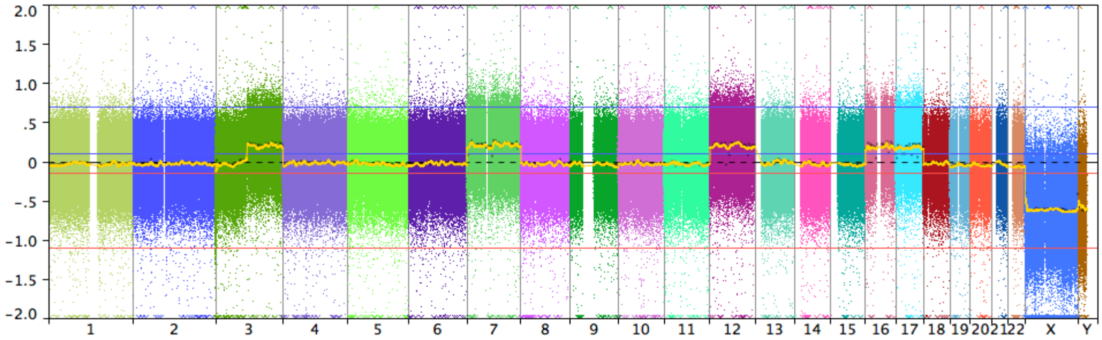

P07

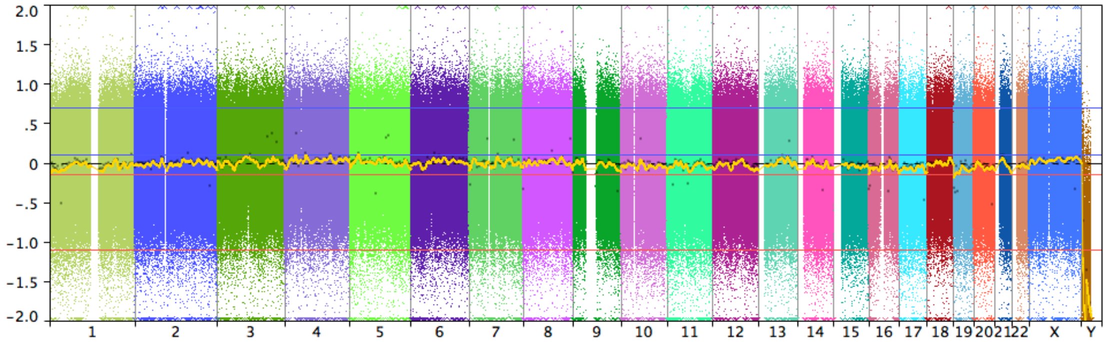

P08

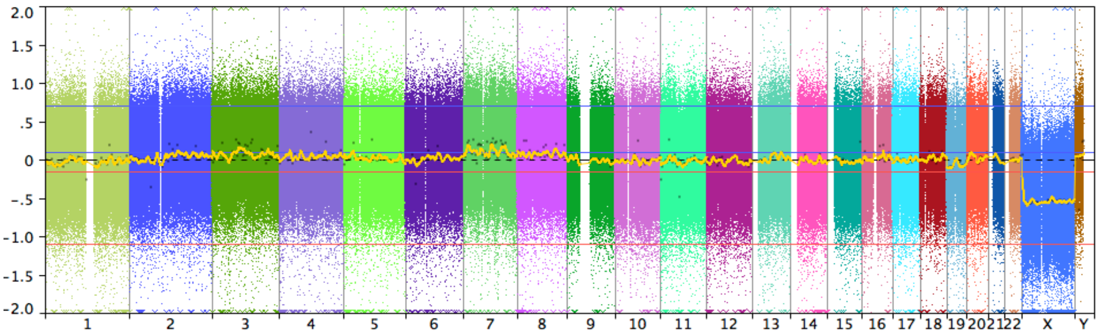

P09

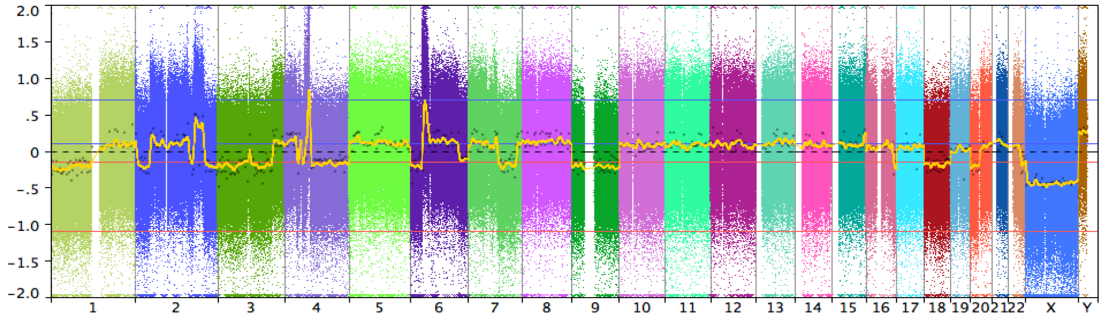

P10

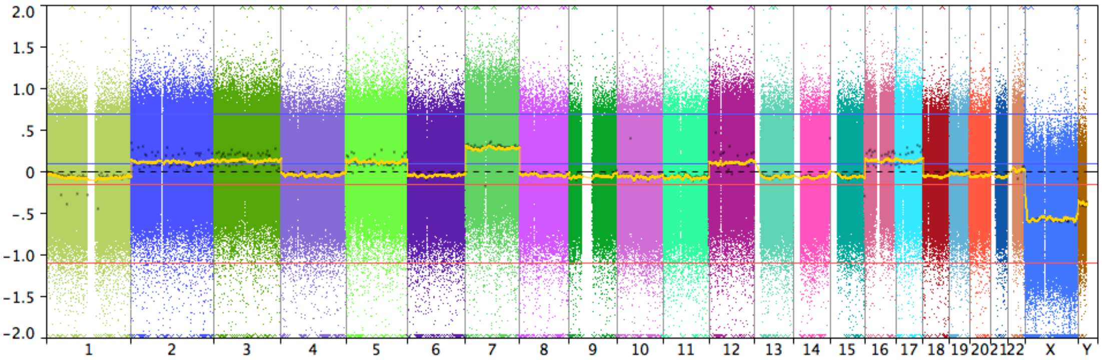

P11

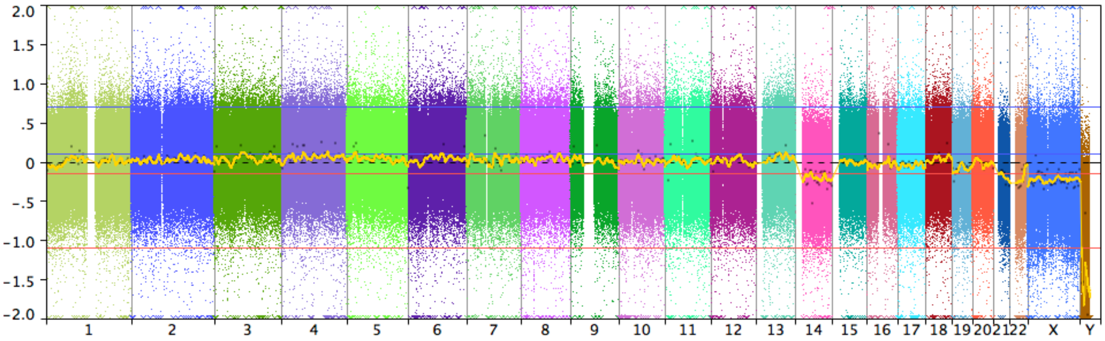

P12

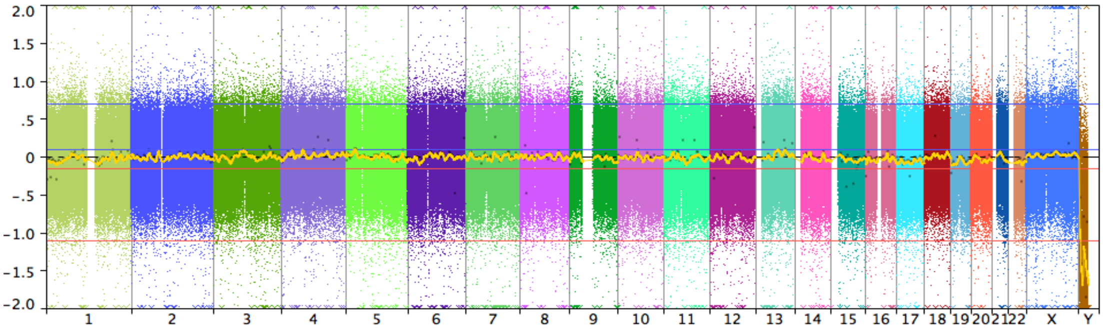

P13

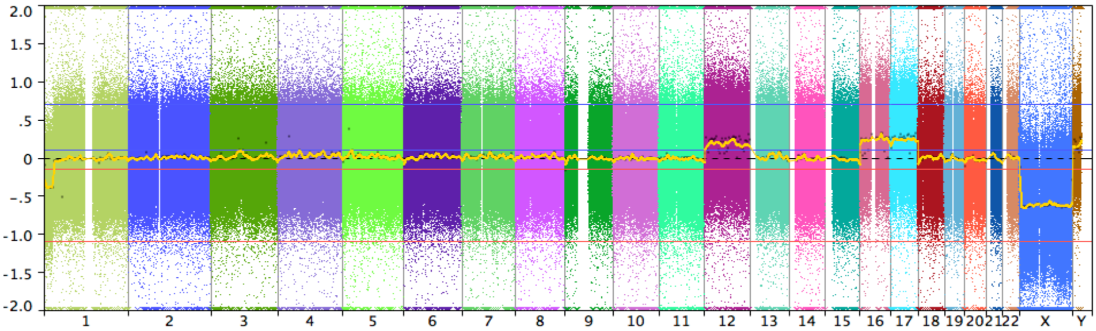

P14

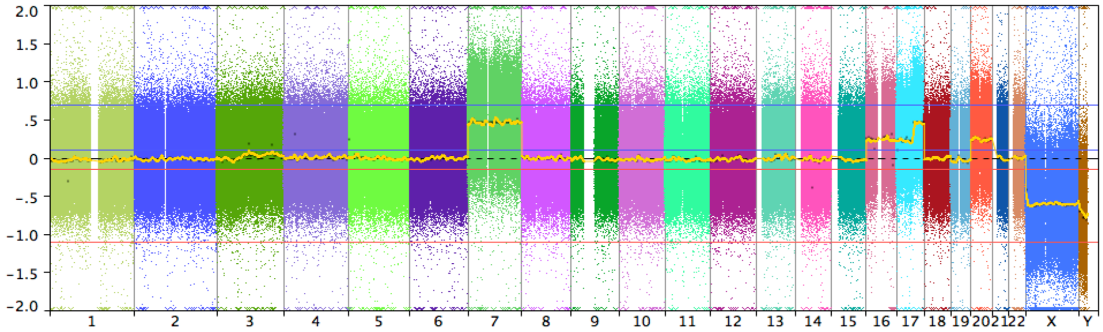

P15

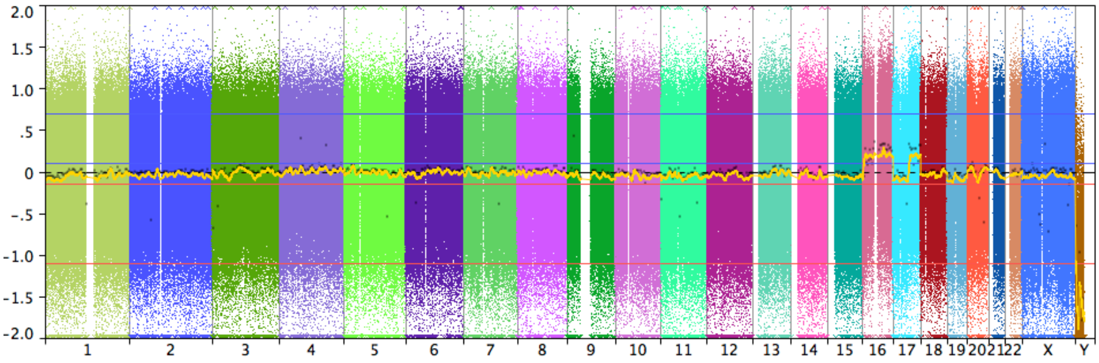

P16

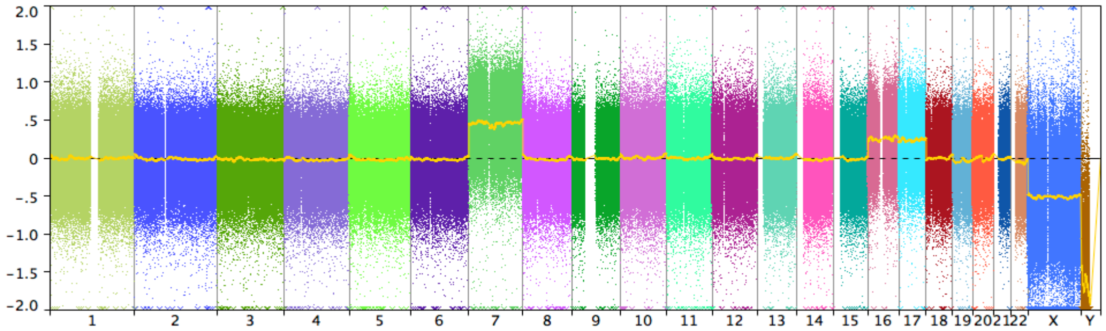

P17

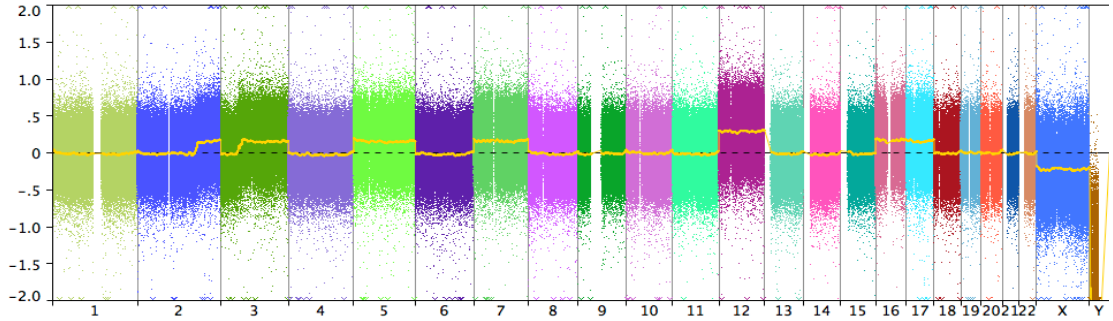

P18

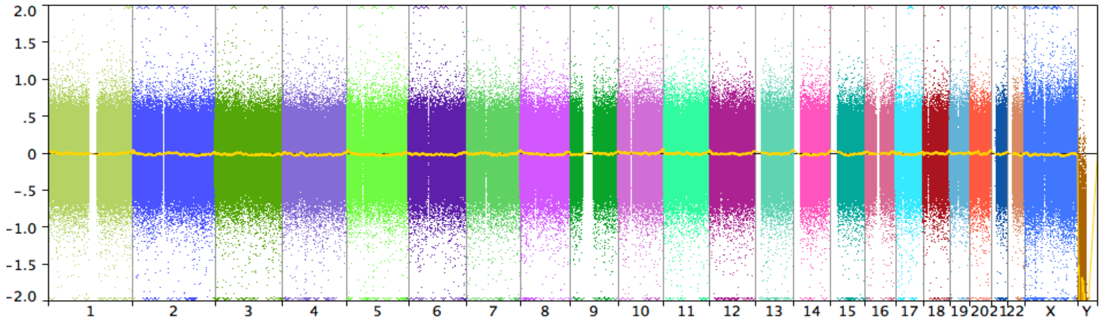

P19

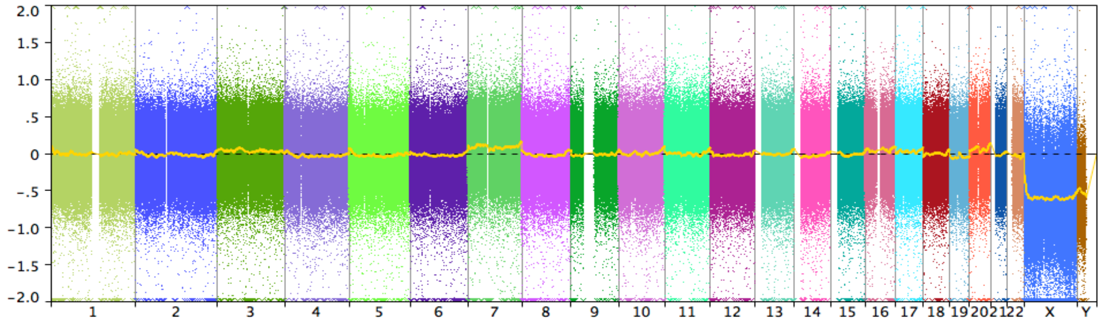

P20

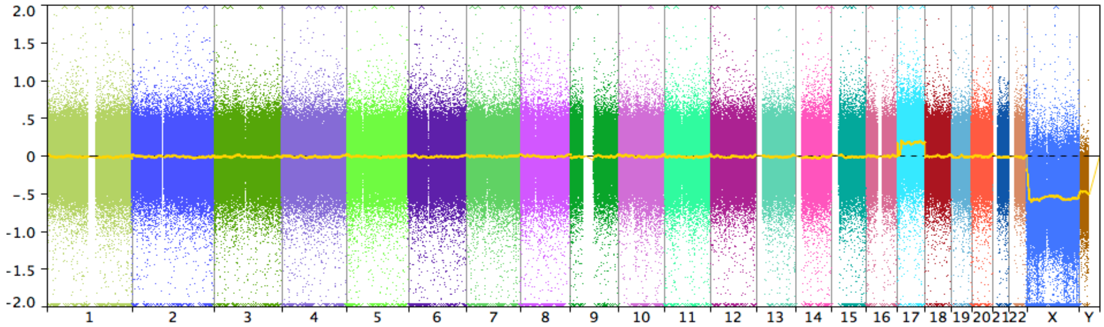

P21

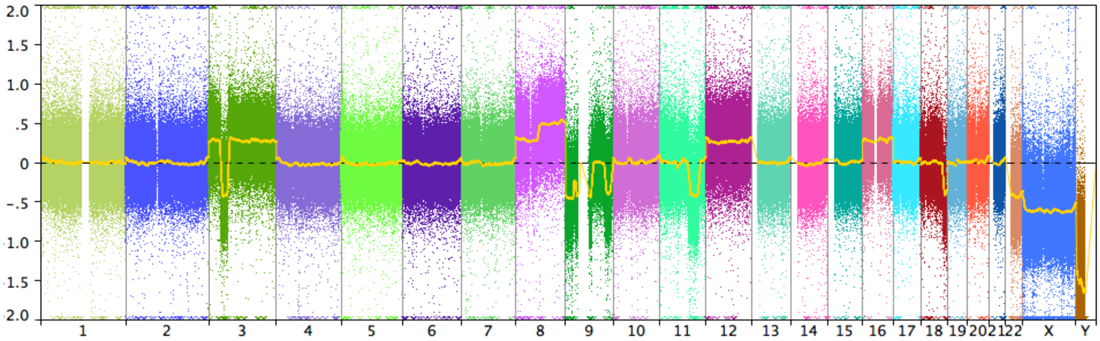

P22

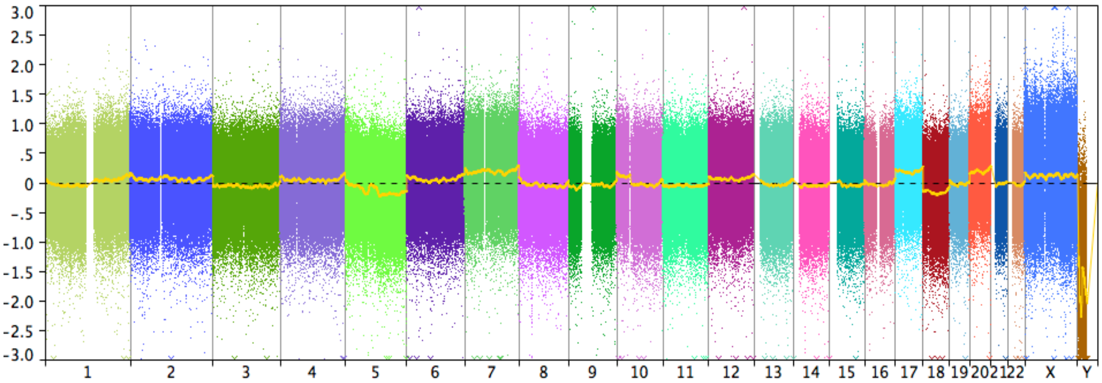

P23

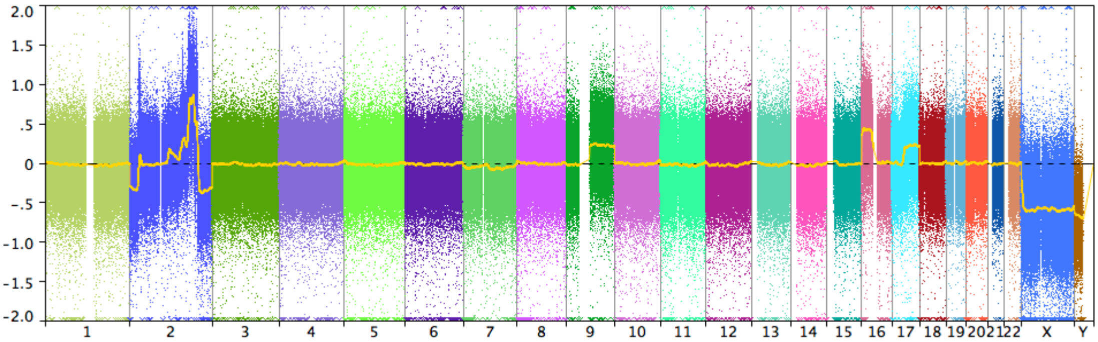

(ii) SCNAs by chromosome (x axis) in the 23 cancers with SNP array data relative to modal copy number (y axis, log scale).

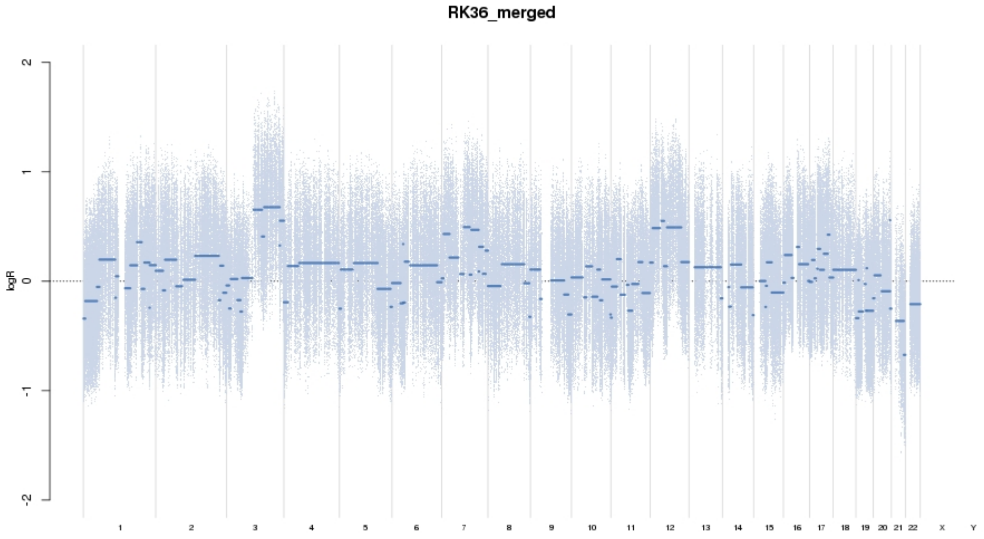
RK36_merged
logR
2
1
0
-1
-2
1 2 3 4 5 6 7 8 9 10 11 12 13 14 15 16 17 18 19 20 21 22 X Y

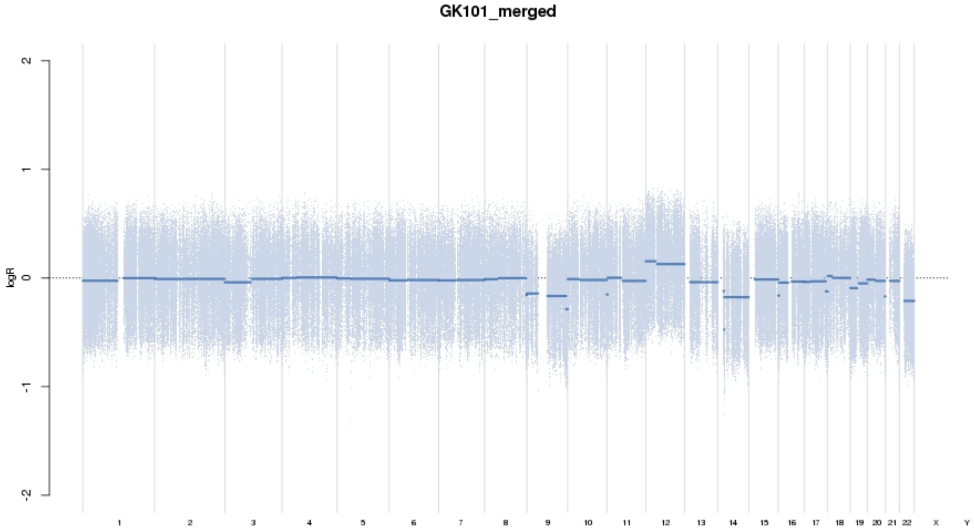
GK101_merged
logR
2
1
0
-1
-2
1 2 3 4 5 6 7 8 9 10 11 12 13 14 15 16 17 18 19 20 21 22 X Y

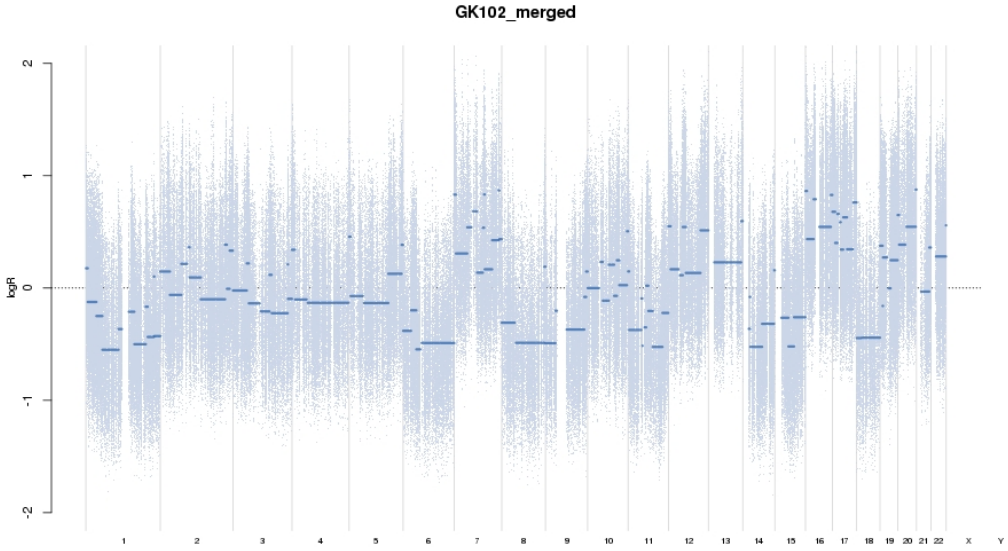
GK102_merged
logR
2
1
0
-1
-2
1 2 3 4 5 6 7 8 9 10 11 12 13 14 15 16 17 18 19 20 21 22 X Y

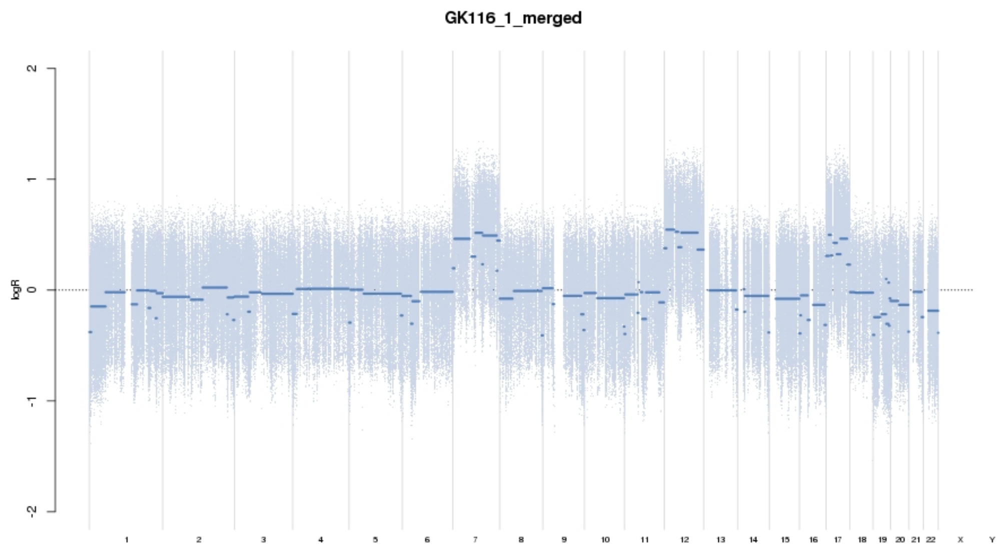
GK116_1_merged
logR
2
1
0
-1
-2
1 2 3 4 5 6 7 8 9 10 11 12 13 14 15 16 17 18 19 20 21 22 X Y

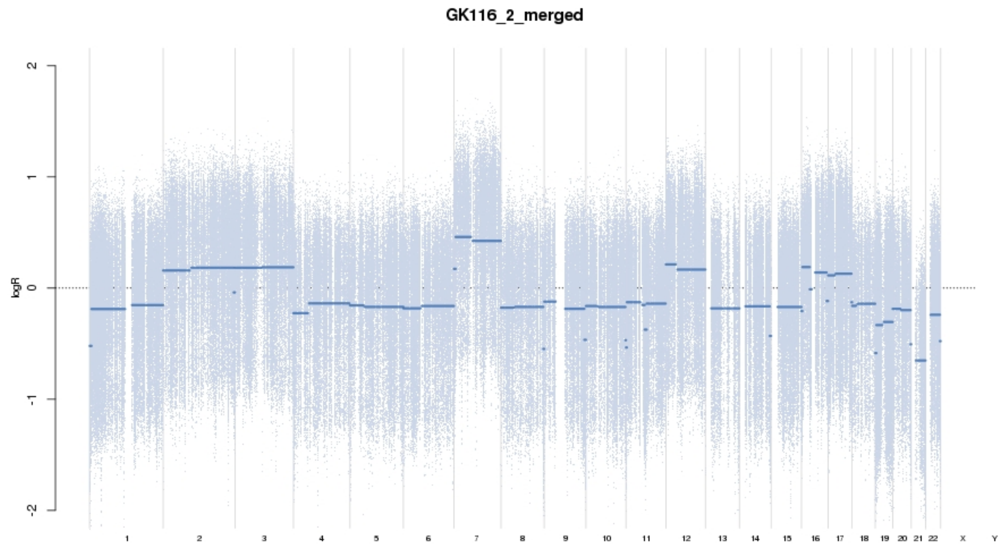
GK116_2_merged
logR
2
1
0
-1
-2
1 2 3 4 5 6 7 8 9 10 11 12 13 14 15 16 17 18 19 20 21 22 X Y

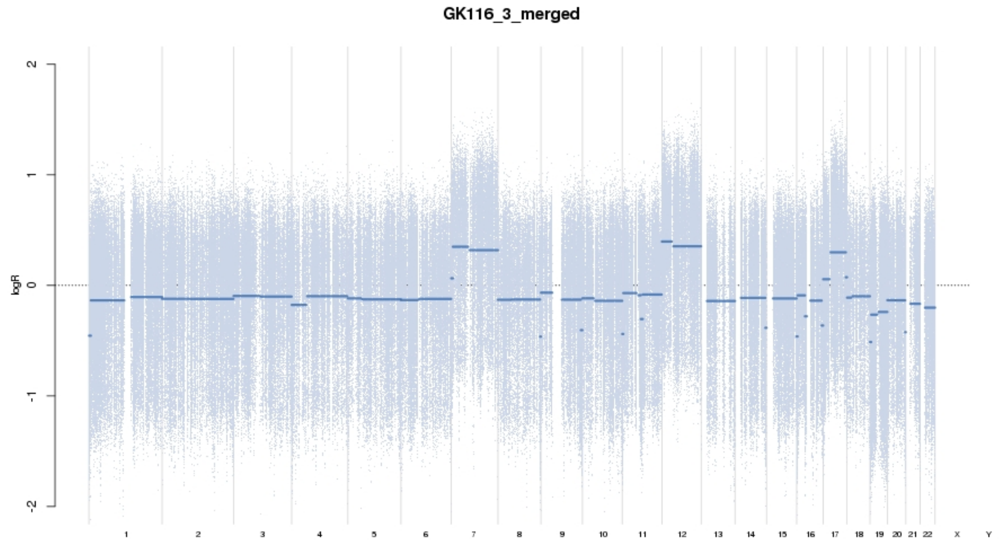
GK116_3_merged
logR
2
1
0
-1
-2
1 2 3 4 5 6 7 8 9 10 11 12 13 14 15 16 17 18 19 20 21 22 X Y

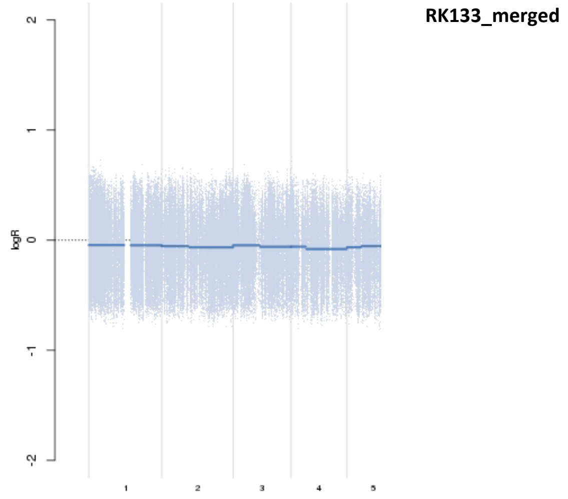

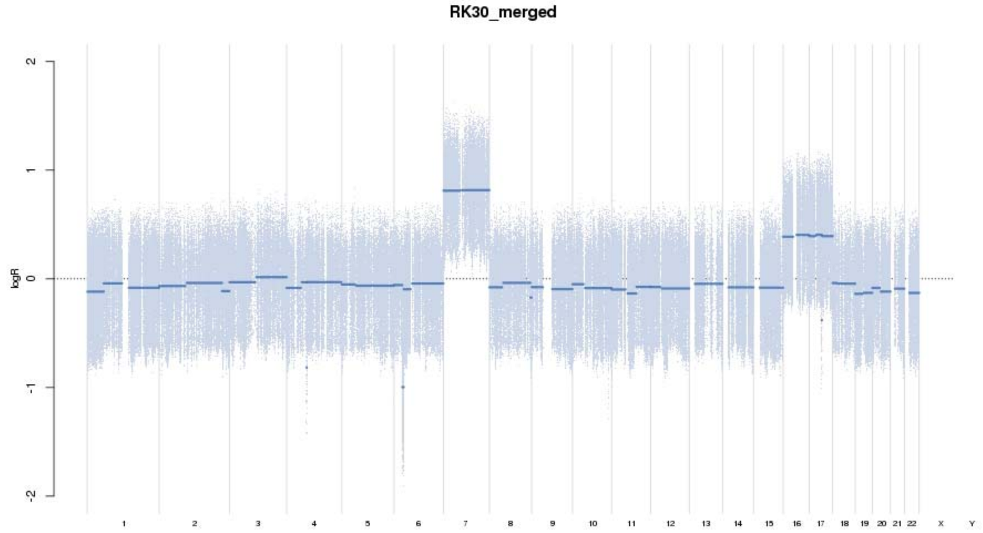

(iii) SCNAs derived from exome sequencing data by chromosome (x axis) in the 8 cancers without SNP array data relative to modal copy number (y axis, log scale). The regional data from the MSeq cancers have been merged for the purposes of this analysis (see also Supplementary Figures 8 and 9).

Supplementary Figure 5. Focal deletions (purple bars) of chromosome 3p involving SETD2 and *BAP1 in pRCC P17.*

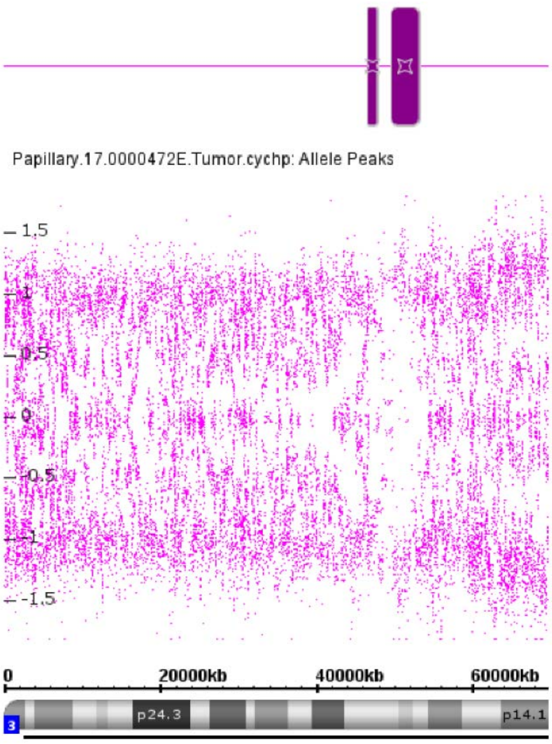

Output is from Affymetric Chromosome Analysis Suite.

Supplementary Figure 6. Rearrangements of chromosomes 4 (upper) and 6 (lower) in pRCC P09 resulting from reciprocal interchanges of material.

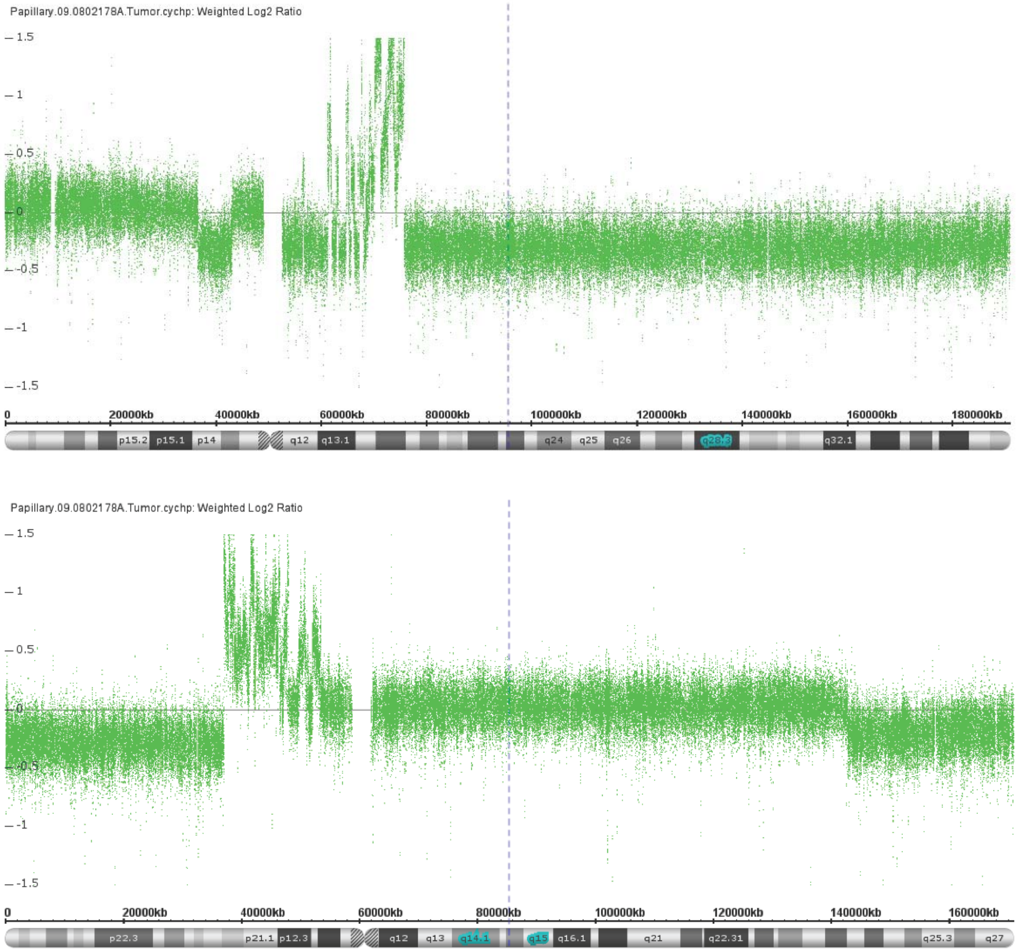

Copy number along each chromosome is shown. Breakpoints are shown in Supplementary Table 6.

Supplementary Figure 7. Deletion extending from the 1p telomere into intron 4 of ARID1A in cancer P13.

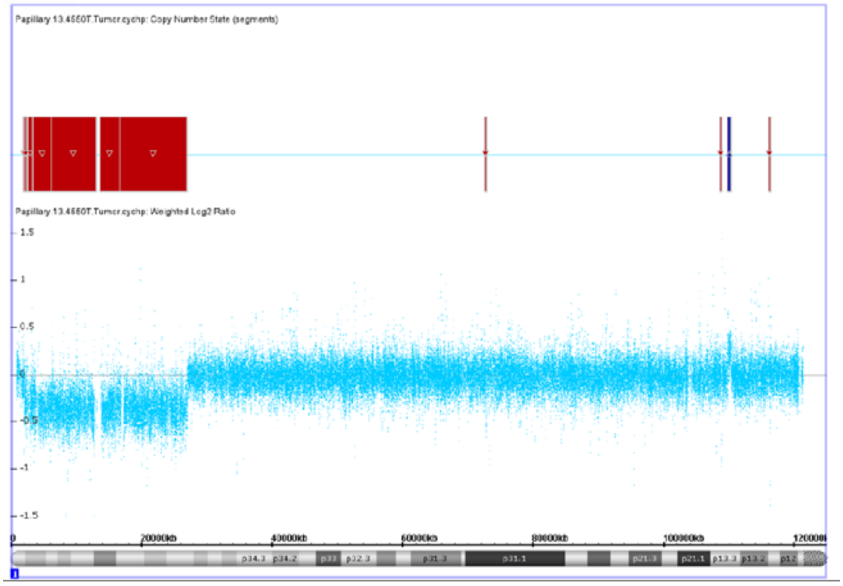

The inversion identified from split sequencing reads extends from the end of the deletion within *ARID1A* to a small region of gain at about 110Mb.

Supplementary Figure 8. Pyclone analysis.

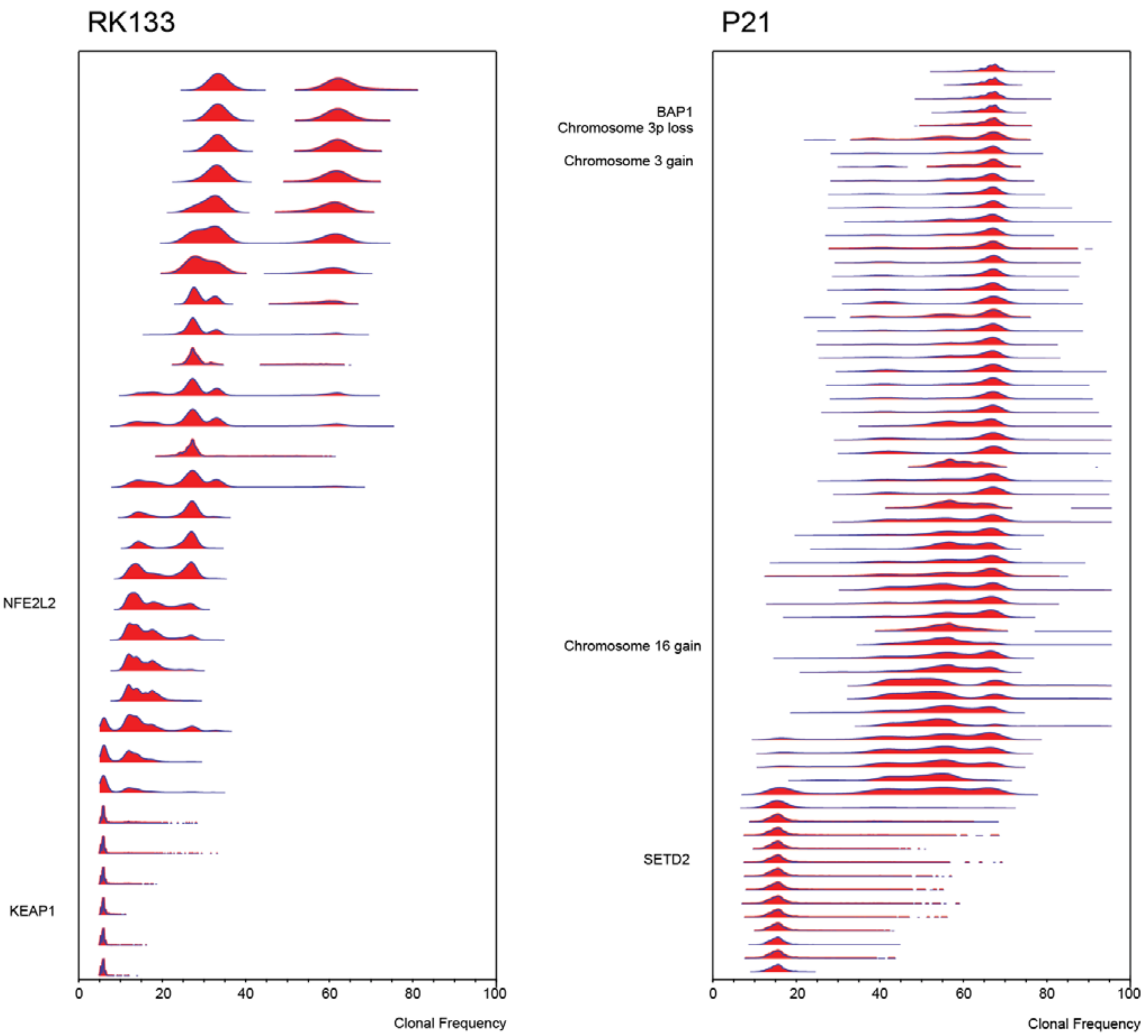

Illustrative Pyclone outputs are shown, with clonal frequency on the x axis and a range of somatic mutations on the y axis. RK133 is a cancer with two Nrf2 pathway SNVs, in *KEAP1* and *NFE2L2.* These mutations are predicted lie within minor sub-clones and Pyclone cluster plots (not shown) show that these mutations do not cluster together, suggesting that they are in separate clones. In P21, the *BAP1* mutation and 3p deletion are in the major clone, with *SETD2* mutation in a sub-clone. Note that for cancers without SNP array data, copy number gains on chromosomes 7, 16 and 17 were identified by VarScan2, providing sufficient resolution to identify copy number changes at the level of the chromosome arms, but perhaps missing smaller changes. For each of the most common large SCNAs (gains of chromosomes 7, 16, 17 and 12, and deletions of chromosomes 3p, 18 and X), a surrogate SNV with copy number-corrected variant allele frequency was produced for use in the Pyclone analysis.

Supplementary Figure 9. Genome-wide Somatic Copy Number profiles of regions of M-seq cancers.

RK30

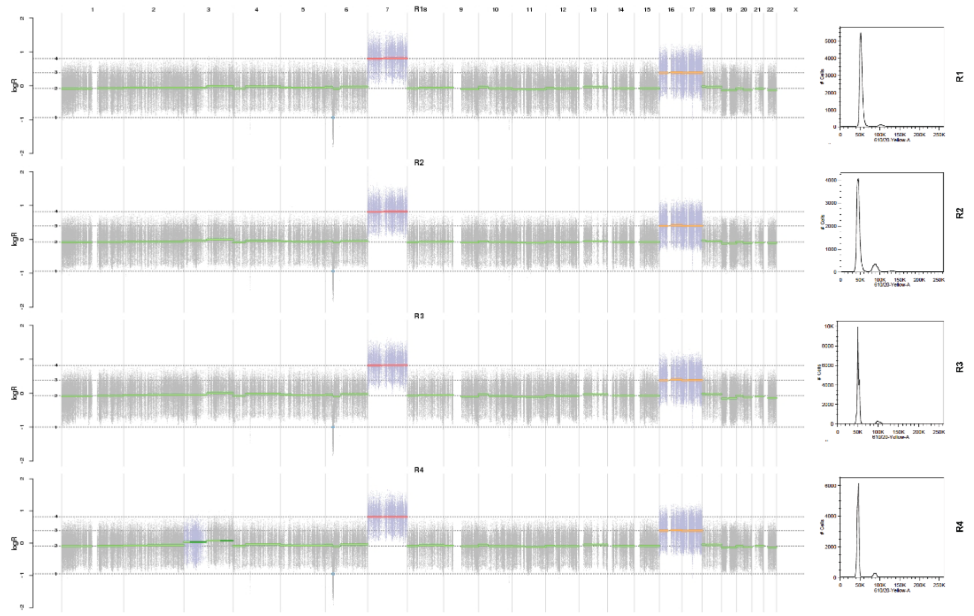

GK116

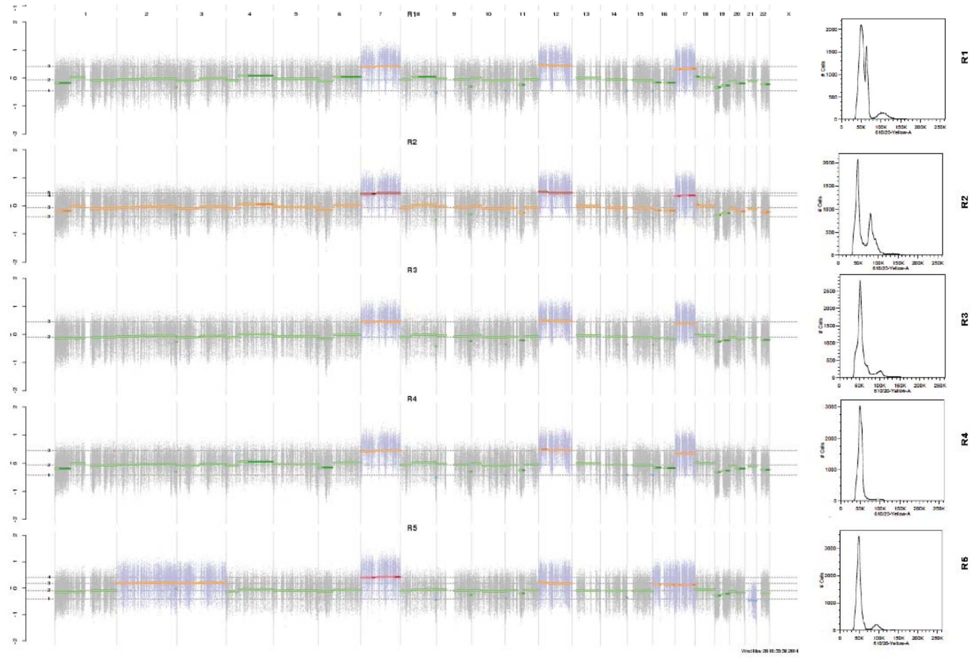

GK101

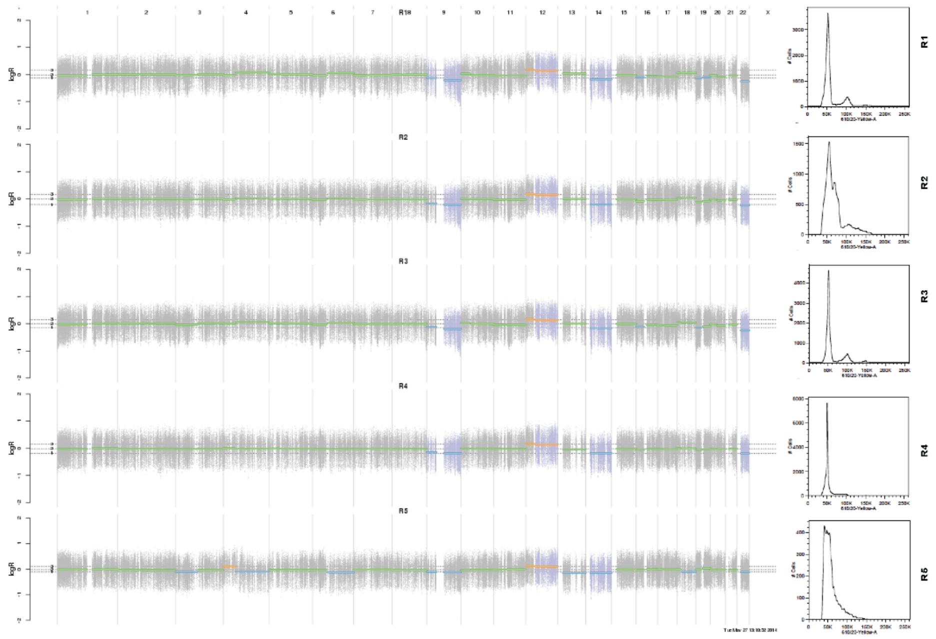
R1
R2
R3
R4
R5

RK36

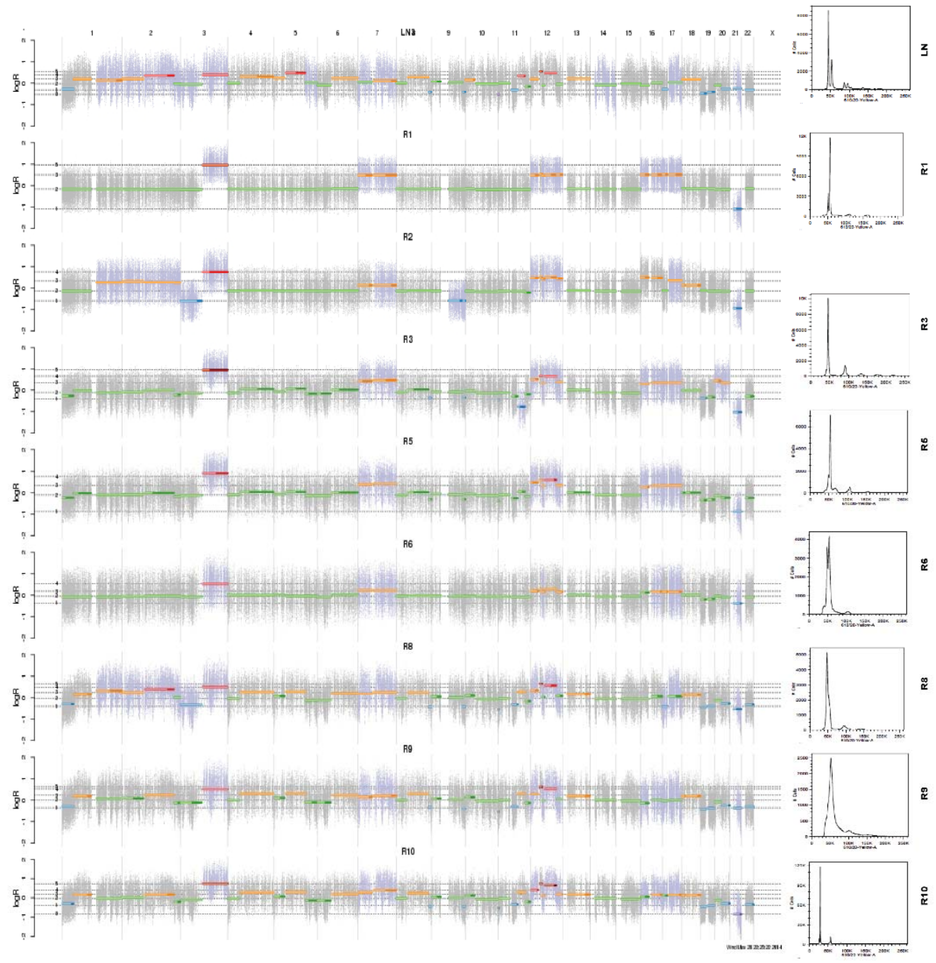
LN
R1
R2
R3
R5
R6
R8
R9
R10

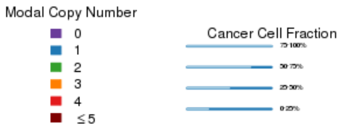
Modal Copy Number
0
1
2
3
4
≤5
Cancer Cell Fraction

For each region of a cancer, panels represent the log-base-2 (logR) of the tumor/normal depth ratio, with genomic segments of constant logR indicated by horizontal lines coloured by integer copy number (see key). Individual logR values are shaded according to the segment's mirrored B-Allele Frequency (mBAF), which in turn reflects loss-of-heterozygosity; in order to identify segments of significant alleleic imbalance, the mBAF distributions within a segment between reference and tumour sample were compared using the Wilcoxon Signed Rank Test with subsequent Bonferroni correction, and segments with $p < 5e-10$ were shaded purple. A segment's predicted 'cancer cell fraction' (clonality) is also indicated. Dashed horizontal lines denote logR thresholds that correspond to clonal integer copy-numbers, as estimated by ABSOLUTE. Adjacent to each panel is the corresponding FACS plot, where y axis represents number of cells and x axis Propidium Iodide staining.

Supplementary Figure 10. Allele-specific analysis of heterozygous single-nucleotide polymorphisms in GK116 and RK36 (haplotype analysis).

GK116

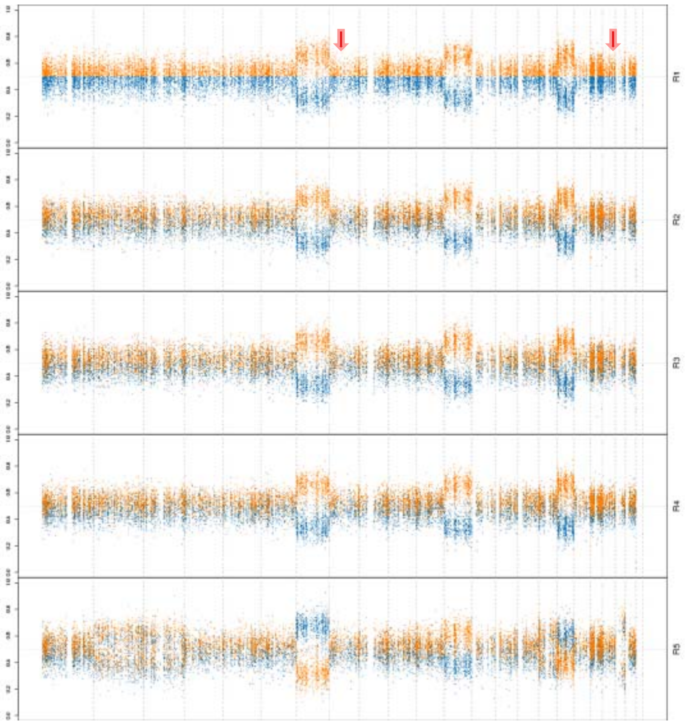

RK36

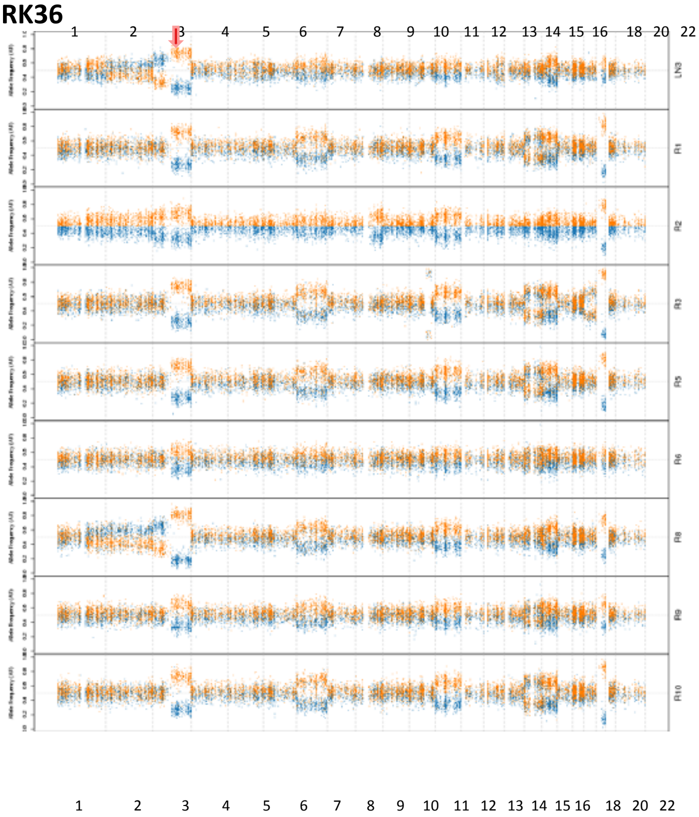

SNP alleles were classed as “major” (AF > 0.5, orange) or “minor” (AF < 0.5, blue) using R1 as a reference. Patterns of major/minor allele frequency were then compared to this region. The gains of chromosomes 7 and 17 appear to involve alternate haplotypes in R1-4 (tumour RK116_1) compared to R5 (tumour RK116_2), which suggests that these gains occurred independently, consistent with independent origins of these multi-tumour foci. (Analysis of R6 (RK116_3) was not successful.) For RK36, deletion of 3p (red arrow) involves alternate haplotypes in R2 compared to R8 and LN3.

Supplementary Figure 11. SETD2 mutation p.Glu1667X in RK36 LN shown in the IGV.

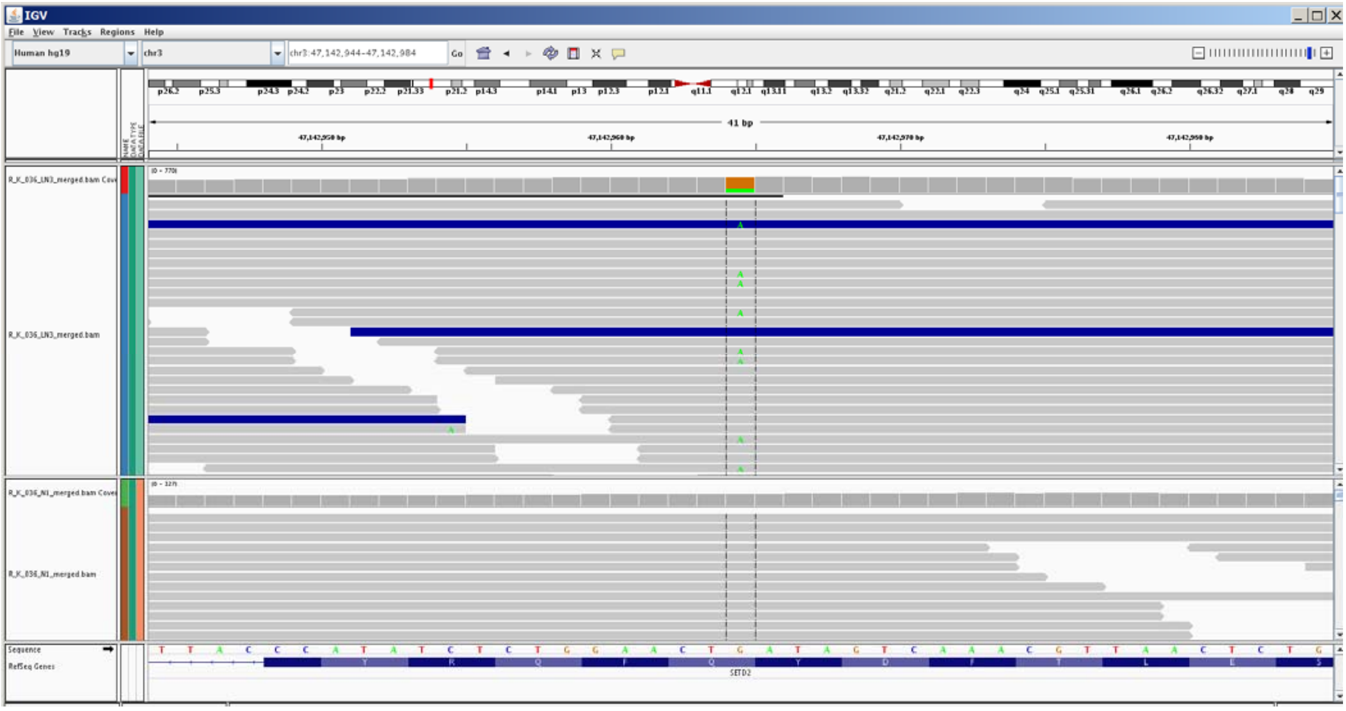

LN is upper, paired normal lower. This mutation was not called by CAVEMAN, although investigation showed that it passed calling and quality control filters, and was therefore a false negative.

Supplementary Tables

Supplementary Table 1. Exome-wide mutation data for each cancer

Summary mutation data include: proportion of substitution mutations of each type; number of high-quality exonic SNVs called; false discovery rate (q) for deviation of mutation spectrum from expected; dN/dS; and Ts:Tv. For the multi-region cancers, only the region with the highest mutation burden is displayed in order to provide a comparison with the other tumours.

Sample	C:G>A:T	C:G>G:C	C:G>T:A	T:A>A:T	T:A>C:G	T:A>G:C	q.value.weighted	No. SNVs	dN/dS	Ts:Tv
RK36	0.130	0.174	0.217	0.000	0.217	0.261	0.12409464	23	1.667	0.366
RK133	0.188	0.101	0.275	0.087	0.159	0.188	0.221522278	69	2.500	0.592
P19	0.000	0.000	0.200	0.000	0.400	0.400	0.136178196	5	0.625	1.117
GK116_3	0.291	0.036	0.291	0.109	0.200	0.073	0.183676611	55	1.565	1.253
P08	0.167	0.100	0.367	0.067	0.200	0.100	0.304649059	30	3.000	1.693
GK116_2	0.139	0.069	0.264	0.153	0.278	0.097	0.211157376	72	3.349	1.486
P20	0.143	0.208	0.260	0.091	0.130	0.169	0.0841801	77	3.204	0.718
GK116_1	0.207	0.017	0.259	0.172	0.276	0.069	0.074128847	58	5.917	1.020
P03	0.136	0.136	0.364	0.068	0.250	0.045	0.208890339	44	1.975	1.076
P01	0.178	0.111	0.422	0.067	0.178	0.044	0.21382186	45	1.723	0.776
GK101	0.200	0.189	0.189	0.100	0.156	0.167	0.047478779	90	3.510	0.669
P11	0.190	0.095	0.333	0.111	0.190	0.079	0.263085016	63	3.676	1.323
P18	0.141	0.172	0.375	0.078	0.141	0.094	0.215528414	64	4.222	1.045
P16	0.119	0.119	0.262	0.167	0.238	0.095	0.137106347	42	3.457	1.271
P05	0.000	1.000	0.000	0.000	0.000	0.000	0.074087074	2	0.500	5.111
P13	0.202	0.138	0.298	0.106	0.149	0.106	0.243415837	94	2.557	0.890
P23	0.200	0.158	0.347	0.074	0.126	0.095	0.201484468	95	3.317	0.897
P15	0.241	0.120	0.337	0.096	0.120	0.084	0.167488319	83	2.557	0.655
P10	0.169	0.104	0.312	0.104	0.247	0.065	0.192535104	77	2.190	0.942
P14	0.169	0.169	0.338	0.085	0.197	0.042	0.164153808	71	1.985	0.973
P02	0.158	0.158	0.456	0.070	0.088	0.070	0.105049345	57	2.356	0.818
P06	0.161	0.195	0.264	0.115	0.172	0.092	0.114029652	87	2.672	0.577
P09	0.241	0.114	0.342	0.051	0.215	0.038	0.112501097	79	3.950	1.060
P22	0.173	0.160	0.320	0.120	0.187	0.040	0.124362865	75	2.896	0.954
P04	0.127	0.073	0.418	0.000	0.309	0.073	0.029660732	55	1.750	1.891
P12	0.087	0.065	0.652	0.065	0.087	0.043	0.000719163	46	1.283	2.154
P21	0.250	0.090	0.240	0.170	0.210	0.040	0.000793532	100	2.463	0.711
GK102	0.106	0.068	0.515	0.030	0.174	0.106	0.000521279	132	1.617	1.591
P17	0.111	0.095	0.440	0.049	0.218	0.086	0.000877165	243	1.678	1.773
P07	0.643	0.054	0.178	0.027	0.065	0.032	1.85E-49	185	1.869	0.241
RK30	0.052	0.076	0.052	0.008	0.111	0.700	1.27E-279	199	3.128	0.208
Mean	0.172	0.141	0.309	0.079	0.183	0.116		78	2.553	1.156
Max	0.643	1.000	0.652	0.172	0.400	0.700		243	5.917	5.111
Min	0.000	0.000	0.000	0.000	0.000	0.000		2	0.500	0.208
Median	0.167	0.111	0.312	0.078	0.187	0.084		71	2.500	0.973

Supplementary Table 2. Germline MET and FH variants in our 31 patients.

Most discovery phase samples had been supplied in an anonymised fashion and no case had been reported as being diagnosed with HPRCC, HLRCC or BHD. After screening *MET, FH, FLCN* and *VHL,* we found the germline variants shown below. All of the *MET* changes are rare variants of doubtful pathogenic effect (http://www.genomed.org/lovd2/home.php?select_db=MET), although the *FH* variant occurs 6 times in the *FH* database and is likely to be pathogenic (http://chromium.liacs.nl/lovd_sdh/variants.php?action=search_unique&select_db=FH).

**Sample ID**	**Gene**	**DNA change**	**Protein change**
P07	MET	c.3029C>T	p.Thr1010Ile
P23	MET	c.504G>T	p.Glu168Asp
RK30	MET	c.1621T>G	p.Cys541Gly
P11	FH	c.1189G>A	p.Gly397Arg

Supplementary Table 3. BAP1, SETD2 and ARID2 mutations found in the 60 replication samples.
All detected somatic changes are shown. Note that for *SETD2,* only the SET, WW and SRI domains – representing moderate mutation hotspots – were screened. N/A=not applicable. SIFT and Polyphen2 functional prediction scores are also shown. Other column headings are as per Tables 1 and 2. Presence of the mutations in the COSMIC v70 (http://cancer.sanger.ac.uk/cosmic/) and TCGA (http://www.cbioportal.org/) on 20th October 2014 is also shown.

ID	Age	Sex	Type	Grade	Gene	Mutation type	Mutation	SIFT	PP2	COSMIC	TCGA
0402014 C	56	M	2		BAP1	frameshift deletion	p.L262fs	N/A	N/A	Not reported	Not reported
13103			2			splicing	c.122+5G>C	N/A	N/A	Not reported	Not reported
15998			2			splicing	c.435+1G>A	N/A	N/A	Not reported	Not reported
2977			2			stopgain SNV	Y546X	N/A	N/A	Not reported	Not reported
509						nonsynonymous SNV	A574V	0.21	0.00	Not reported	Not reported
14863/09	72	M	2	3	SETD2	nonsynonymous SNV	G1563V	0.00	1.00	Not reported	Other mutations at same site
9853/07	70	M	2	3		nonsynonymous SNV	G1563V	0.00	1.00	Not reported	Other mutations at same site
36808/05	70	F	1/2	3		nonsynonymous SNV	P2379S	0.07	1.00		
27758/07	55	F	2	3	ARID2	nonsynonymous SNV	A205T	0.18	1.00	Not reported	Not reported
702164 A2	55	M	2			nonsynonymous SNV	P346L	0.24	1.00	Two cancers	Not reported
23179/06	65	F	2	4		frameshift deletion	p.G1605fs	N/A	N/A	Not reported	Not reported

Supplementary Table 4. Intogen (a) and Mutsig (b) analysis of genes with an over-representation of somatic SNVs in our full pRCC set.

Genes with nominally significant over-representation (p<0.05) are shown. Note that these data are not filtered for predicted functional effects or visual IGV inspection (except for putative driver genes highlighted in Table 2). Standard Intogen and MutSigCV annotation is used. For Intogen, SAMPLE_FREQ represents the number of samples from the SAMPLE_TOTAL with somatic SNVs in each gene, with SAMPLE_PROP showing the proportion of mutated samples. For MutsigCV, p and q values for each gene are shown.

(a)

SYMBOL	FM_PVALUE	FM_QVALUE	SAMPLE_FREQ	SAMPLE_TOTAL	SAMPLE_PROP
TTN	0.0002	0.0484	12	31	0.3871
TRIM37	0.0011	0.0884	3	31	0.0968
MLL5	0.0011	0.0884	5	31	0.1613
CUBN	0.0033	0.1689	5	31	0.1613
TBC1D9B	0.0043	0.1689	5	31	0.1613
BAP1	0.0043	0.1689	2	31	0.0645
MYO1B	0.0090	0.2333	4	31	0.1290
ACACB	0.0126	0.2333	5	31	0.1613
LAMB2	0.0133	0.2333	3	31	0.0968
S1PR1	0.0217	0.2333	2	31	0.0645
NYNRIN	0.0217	0.2333	2	31	0.0645
DUS3L	0.0217	0.2333	6	31	0.1935
TNFRSF25	0.0217	0.2333	4	31	0.1290
BIRC2	0.0217	0.2333	3	31	0.0968
RBM42	0.0217	0.2333	3	31	0.0968
NAV2	0.0217	0.2333	3	31	0.0968
MACF1	0.0217	0.2333	3	31	0.0968
KIAA2018	0.0217	0.2333	2	31	0.0645
NUDT16	0.0217	0.2333	2	31	0.0645
ZBTB20	0.0217	0.2333	3	31	0.0968
ARID2	0.0217	0.2333	4	31	0.1290
GOLGB1	0.0217	0.2333	2	31	0.0645
KHSRP	0.0237	0.2433	3	31	0.0968
PABPC1	0.0272	0.2569	4	31	0.1290
PDLIM2	0.0275	0.2569	3	31	0.0968
FLII	0.0289	0.2569	3	31	0.0968
PTPRK	0.0300	0.2569	5	31	0.1613
P2RX7	0.0305	0.2569	3	31	0.0968
SLC22A18	0.0353	0.2872	2	31	0.0645
SIK3	0.0410	0.3222	2	31	0.0645
SPAG1	0.0432	0.3288	4	31	0.1290
SDCCAG3	0.0449	0.3310	2	31	0.0645

(b)

gene	p	q
C2orf66	1.21E-04	1
HEXIM1	9.96E-03	1
GHRH	1.49E-02	1
C4orf26	1.99E-02	1
PABPC1	2.00E-02	1
FDX1L	2.18E-02	1
GDPD3	2.73E-02	1
FAM19A3	3.19E-02	1
SAT2	3.24E-02	1
KHSRP	3.29E-02	1
TMEM54	3.33E-02	1
APOF	3.59E-02	1
BAMBI	3.97E-02	1
KIRREL	4.08E-02	1
NECAP1	4.14E-02	1
PPFIBP2	4.28E-02	1
PDLIM2	4.98E-02	1

Supplementary Table 5. Intogen (a) and Mutsig (b) analysis of TCGA data SNVs in 20 potential driver from our pRCCs (see Figure 3).

See Supplementary Table 3 for further details of abbreviations. Note that the q values are for genome-wide analysis, not for our set of 20 genes only. The 20 gene set-specific q=0.05 is equivalent to p=0.02 for Intogen and p=0.011 for Mutsig. Note the support for *SETD2, BAP1, ARID2* and the Nrf2 pathway genes as drivers, together with several other of the 20 genes, such as *TRIO*, although the two programs show some discordance.

(a)

SYMBO	FM_PVALUE	FM_QVALUE	SAMPLE_FREQ	SAMPLE_TOTAL	SAMPLE_PROP
CUL3	2.11E-12	2.68E-09	7	172	4.1%
SETD2	6.25E-08	2.26E-05	13	172	7.6%
NFE2L2	7.60E-05	0.009629706	6	172	3.5%
BAP1	0.000925348	0.045977074	8	172	4.7%
TRIO	0.004285294	0.081753501	7	172	4.1%
TRIM37	0.014321377	0.180549099	2	172	1.2%
ARID1A	0.02451482	0.22737983	9	172	5.2%
CNOT1	0.03814206	0.285109084	7	172	4.1%
ARID2	0.03921678	0.289346423	4	172	2.3%
CDKN2A	0.292248528	0.822266213	3	172	1.7%
MED13	0.351724883	0.927441055	3	172	1.7%
CUBN	0.597600234	1	13	172	7.6%
AKAP9	0.731783187	1	3	172	1.7%
KEAP1	0.759246255	1	2	172	1.2%
PLEC	0.999999918	1	9	172	5.2%
MET			13	172	7.6%
RADIL			3	172	1.7%
FH			2	172	1.2%
CCDC168			1	172	0.6%
FLCN			1	172	0.6%

(b)

gene	p	q
SETD2	3.57E-04	3.54E-01
CUL3	1.19E-03	6.94E-01
BAP1	4.49E-03	1
MET	1.04E-02	1
CDKN2A	1.86E-02	1
FH	1.96E-02	1
ARID1A	5.19E-02	1
NFE2L2	1.25E-01	1
TRIM37	3.51E-01	1
ARID2	3.68E-01	1
KEAP1	3.70E-01	1
CNOT1	5.73E-01	1
FLCN	7.09E-01	1
TRIO	9.03E-01	1
MED13	9.62E-01	1
RADIL	9.64E-01	1
PLEC	1.00E+00	1
CUBN	1.00E+00	1
AKAP9	1.00E+00	1

Supplementary Table 6. Regions of the most common pRCC copy changes in the 23 tumours with SNP array data. Note that P07 and P12 also had copy-neutral LOH involving a large region around *BAP1* and *PBRM1*. P17 had two focal deletions involving *BAP1* and *SETD2*. Locations of the relevant chr3p genes are: *SETD2*, chr3:47,057,898-47,205,467; *PBRM1*, chr3:52,579,368-52,719,866, *BAP1*, chr3:52,435,020-52,444,121; *VHL*, chr3:10,183,319-10,195,354; and *MET,* chr7:116,312,459-116,438,440.

Tumour	3p deletion	7 gain	12 gain	16 gain	17q gain	18 deletion
P01	All	No	No	No	All	No
P02	All	All	0-34	No	No	All q
P03	No	All	No	All	All	No
P04	No	No	No	No	No	No
P05	No	All	No	All	All	No
P06	No	All	All	All	All	No
P07	No	No	No	No	No	No
P08	No	All	No	No	No	No
P09	All	No	No	No	No	All
P10	No	All	All	All	All	No
P11	No	No	No	No	No	No
P12	No	No	No	No	No	No
P13	No	No	All	All	All	No
P14	No	All	No	All	All	No
P15	No	No	No	5Mb-ter	46Mb-ter	No
P16	No	All	No	All	All	No
P17	46.7-47.8, 49.7-53.3	All	All	All	All	No
P18	No	No	No	No	No	No
P19	No	All	No	No	No	No
P20	No	No	No	No	All	No
P21	36.6-58.2Mb	No	All	All	No	66Mb-ter
P22	All	All	All	No	All	All
P23	No	No	No	ter-34.2Mb	33Mb-ter	No

Supplementary Table 7. Small regions of copy number change in the 23 PRCCs with SNP array data.

Changes are shown by tumour and location, together with examples of genes within the region. The last column indicates whether the presence of the change was supported in Complete Genomics data (N/A=not available).

**Sample**	**Deletion or gain**	**Chr**	**Start (Mb)**	**End (Mb)**	**Example genes**	**Present in CG data**
P02	Deletion	9	21.9	22.0	CDKN2A	N/A
P05	Deletion	10	110.2	111.2	DA754482	N/A
P08	Gain	4	26.8	27.7	None	Not found
P10	Deletion	4	134.9	135.4	PABPC4L	Yes
P10	Gain	10	60.4	61.1	PHYLIPL, FAM13C	Yes
P11	Gain	7	6.4	7.4	RAC1, ZDHHC4, RPA3	Yes
P13	Gain	20	2.5	3.4	IDH3B, PTPRA, EBF4	Not found
P14	Deletion	16	78.3	78.6	WWOX	Yes
P14	Deletion	19	20.4	20.8	ZNF737, ZNF626, ZNF65	Not found
P16	Gain	12	129.0	129.8	FZD10, PIWIL1, RIMBP2	N/A
P16	Gain	21	43.7	44.1	HSF2BP, RRP1B, PDXK	N/A
P21	Gain	22	32.2	32.6	MIR650	N/A

Supplementary Table 8. Summary of predicted clonality in single-sample pRCCs.
The table shows whether putative driver mutations and the most common somatic SCNAs (gains of chromosomes 7, 12, 16 or 17) are predicted to be present in the major clone (“Major”) or sub-clone (“Sub”) of each cancer. Data are derived from single-sample Pyclone analysis except for the Mseq cancers (RK30, RK36, GK101, RK116_1) where data are derived from assessment of each sampled region. Uncertain clonal assessments are shown by “?”. The final column shows whether the major copy number changes are predicted to be present in the main clone for each cancer. Cancers without changes in these genes or chromosomes chromosomes 7, 12, 16 or 17 are not shown.

	BAP1	SETD2	ARID2	NFE2L2	CUL3	KEAP1	7 gain	12 gain	16 gain	17 gain	CN gains in main clone?
P01			Main							Main	Yes
P02	Main						Main			Sub	No
P03							Main		Main	Main	Yes
P05							Main		Main	Main	Yes
P06							Main		Main	Main	Yes
P08							Main		Main		Yes
P10							Main	Main	Main	Main	Yes
P11			Main								
P13						Sub		Main	Main	Main	Yes
P14							Main		Main	Main	Yes
P15			Main						Main	Main	Yes
P16					Main		Main		Main	Main	Yes
P17							Main	?	?	?	?
P19							?				?
P20				Sub						Main	Yes
P21	Main	Sub						Main	Main		Yes
P22							Main			Main	Yes
P23									Main	Main	Yes
RK30							Main		Main	Main	Yes
RK36		Sub									?
GK101								Main			Yes
GK102	Sub										?
GK116_1							Main	Main		Main	Yes
GK116_2		Sub					Main	Main	Main	Main	Yes
GK116_3							Main	Main		Main	Yes
RK133				Sub		Sub	Main		Main	Main	Yes

Supplementary Methods

Further details of basic sequencing performance parameters

In the full set of tumours, a median of 90% of the exome was covered at ≥30X (range 77-95%), with a median of 93% (range 85-96%) covered at ≥20X. For the 8 Complete Genomics samples, whole-genome coverage was very similar to that of the exome (see main text). Overall, there were no significant differences in median exome coverage or somatic single nucleotide variant (SNV) burden among the platforms used (P>0.7, Kruskal-Wallis test). The number of high-quality exonic somatic SNVs called with high confidence ranged from 2 to 243 per cancer (median=73). Two cancers had fewer than 10 somatic SNVs, but both of these had good coverage and were subsequently found to have somatic copy number alterations (SCNAs), thus excluding problems arising from a low proportion of cancer cells in the samples. There was no association between sequencing depth and number of SNVs (P=0.57, linear regression). By comparison with the exome data, the whole-genome somatic SNV burden in the Complete Genomics samples ranged from 3589 to 8137 (median=6779).

In contrast to the very similar SNV burdens across platforms, small insertion-deletion (indel) mutations were less frequent in the Complete Genomics data than the Illumina exome data (details not shown). This phenomenon has been reported previously and presumably results from the gapped reads inherent to the Complete Genomics method [1]. We therefore restricted our further analysis of indels to the identification of potential driver mutations.

The exonic transition:transversion (Ts:Tv) ratio compared with the human reference genome was close to 2 in all the paired normal samples. In the cancers, however, wide variation in this measure was observed, with a median ratio close to 1 (Table 1, Supplementary Table 1). With the exception of the two cancers with very few somatic SNVs, the non-synonymous:synonymous somatic SNV ratio (dN/dS) was >1, with a mean of 2.6 (Table 1, Supplementary Table 1). There was a weak positive association between dN/dS and mutation burden (P=0.040, linear regression), but not with Ts:Tv. Neither Ts:Tv nor dN/dS varied significantly among sequencing platforms (P>0.15, Kruskal-Wallis test).

Mutations in *SETD2, BAP1, ARID2, CUL3, NFE2L2* and *KEAP1* were validated in all samples. M-seq cancers additionally underwent extensive technical validation using a custom Ion Torrent panel (content available on request, see Methods). For the remaining 23 pRCCS, technical validation was performed using a 75 cancer gene Ion Torrent panel. This confirmed a total of 103/108 (95%) germline and somatic SNVs and 20/25 (80%) small indels that had been reported by the Illumina or Complete Genomics platforms.

Functional annotation of driver gene mutations and further analysis to search for new driver mutations

BAP1 mutations appeared to be protein-inactivating. They included two frameshift changes, one nonsense mutation, two mutations each affecting the first base of a splice donor or acceptor site and hence predicted to cause exon skipping, and one at +5 of a splice site with potential effects on splicing. Two missense mutations were also found. One was a disruptive substitution of an amino acid at codon 185. This residue is evolutionarily-conserved, and lies next to a catalytically-important residue in the BAP1 ubiquitin hydrolase (UCH) domain. It has mixed predicted functional effects (SIFT=0.41, Polyphen2=1.00). The other missense change was at residue 574 (Supplementary Table 2), although there was lower confidence in the pathogenicity of this change (SIFT=0.21, Polyphen2=0.00): the amino acid change is non-disruptive, conservation is limited to vertebrates, and residue 574 is at site of no known special importance for BAP1 function. Chromosome 3p copy number status was assessed in two *BAP1*-mutant cancers (P02, P21), and both showed deletion around *BAP1*, consistent with a tumour suppressor gene (Supplementary Table 3).

Most *SETD2* mutations also appeared to be protein-inactivating. One cancer (RK36) had acquired two nonsense mutations and a frameshift change. Another cancer, P21 – which also had a *BAP1* mutation and 3p deletion – had a complex *SETD2* change that we determined to be a 52bp frameshift deletion by inspection of sequencing reads and re-mapping to the gene, followed by Sanger sequencing validation. A third cancer (GK116_2) had the missense *SETD2* change p.Ser791Pro. Although this is disruptive in terms of the amino acid change, missense

mutations at or near this site have been reported very rarely in other cancers, the residue is only conserved in mammals, the region of the protein affected has not known special function and predicted functional effects are modest (SIFT=0.3, Polyphen2=0.001). Three further missense *SETD2* changes were found in the replication samples (Supplementary Table 2), all at highly-conserved sites with predicted strong functional effects: two were the recurrent change p.Gly1563Val change and the others was p.Pro2379Ser.

Four of the 6 *ARID2* mutations were frameshift changes. The remaining two mutations (p.Ala205Thr, p.Pro346Leu) were missense changes at highly conserved residues and with moderate or greater predicted functional effects (Supplementary Table 2), although neither amino acid change was highly disruptive. p.Pro346Leu has previously been reported as a heterozygous change in hepatocellular carcinoma. Five of the 23 cancers assessed showed gain of chromosome 12 including the *ARID2* locus, but none of these 5 was an *ARID2*-mutant tumour. Conversely, the *ARID2* mutations were not accompanied by deletion or LOH, suggesting that they might be haploinsufficient.

Of the other recurrently-mutated pRCC genes, some had *a priori* expectation of functional importance in renal carcinogenesis, including *TRIO, RADIL* and *MED13* (Table 2). *TRIO* is a large gene that acquired missense changes in 4 cancers and encodes a Rho guanine nucleotide exchange factor; *RADIL* is involved in control of cell adhesion and migration; and *MED13* codes for a subunit of the mediator transcriptional coactivator complex.

We examined the 8 cancers with whole-genome data for non-exomic mutations around the genes in Table 2. However, no changes predicted to influence promoter activity, transcription factor binding or targeted to highly conserved regions were found (details not shown).

Formal assessment to detect significantly over-mutated genes and functional pathways was performed on our 31 sequenced cancers using the Intogen [2] and MutSigCV programs [3]. Although MutSigCV did not highlight any of our putative driver genes (Supplementary Table 4a), Intogen provided confirmatory evidence (P<0.05) for the importance of *BAP1, ARID2* and another two of our 12 selected genes, *TRIM37* and *CUBN* (Supplementary Table 4b). All three *TRIM37* mutations were protein-truncating (Table 2), but the gene is poorly characterised. *CUBN* encodes the intrinsic factor-vitamin B12 receptor, which is expressed in the kidney, and mutations were mostly missense changes (Table 2). The roles of both these genes as pRCC drivers require confirmation. We then investigated the top genes in the MutSigCV and Intogen analyses without applying our filter to exclude variants of benign predicted functional effects. However, after other exclusions were applied and reads were inspected in the IGV, no additional genes mutated in 3 or more cancers were found. Intogen pathway analysis suggested an over-representation of mutant genes involved in Metabolic pathways, Pathways in cancer and Endocytosis (details not shown).

Clinicopathological-molecular associations

We assessed associations between molecular data (total SNV number, total chromosomal scale change number, dN/dS, Ts:Tv cluster group, predicted number of clones, gains of chromosomes 7, 16 and 17, outlying SNV mutation spectrum as measured by deviation from spectrum in the whole cancer set) and clinicopathological data (age at presentation, sex, T stage, N stage, Fuhrman grade, Type 1 v 2). The only nominally significant association was between outlying mutation spectrum and T stage (P=0.035, Kruskal-Wallis test). Specifically, chromosome 7 gain was present in many type 2 pRCCs and was not associated with type 1 morphology contrary (P=0.62, Fisher's exact test), to some previous reports that chr7 and/or *MET* gain were specific to type 1 pRCCs. Overall, it remains unclear whether a molecular classifier can substitute for the troubled morphological type 1/2 classifier in pRCCs.

Supplementary References

1. Lam, H.Y. *et al.* Performance comparison of whole-genome sequencing platforms. *Nat Biotechnol* **30**, 78-82 (2012).
2. Gundem, G. *et al.* IntOGen: integration and data mining of multidimensional oncogenomic data. *Nat Methods* **7**, 92-3 (2010).
3. Lawrence, M.S. *et al.* Mutational heterogeneity in cancer and the search for new cancer-associated genes. *Nature* **499**, 214-8 (2013).
